# Urine-Xpert Ultra for the diagnosis of tuberculosis in people living with HIV: a prospective, multicentre, diagnostic accuracy study

**DOI:** 10.1016/S2214-109X(24)00357-7

**Published:** 2024-11-20

**Authors:** Bianca Sossen, Rita Székely, Madalo Mukoka, Monde Muyoyeta, Elizabeth Nakabugo, Jerry Hella, Hung Van Nguyen, Sasiwimol Ubolyam, Berra Erkosar, Marcia Vermeulen, Chad M Centner, Sarah Nyangu, Nsala Sanjase, Mohamed Sasamalo, Huong Thi Dinh, The Anh Ngo, Weerawat Manosuthi, Supunnee Jirajariyavej, Nhung Viet Nguyen, Anchalee Avihingsanon, Andrew D Kerkhoff, Claudia M Denkinger, Klaus Reither, Lydia Nakiyingi, Peter MacPherson, Graeme Meintjes, Morten Ruhwald, FujiLAM Study Consortium, Bianca Sossen, Rita Székely, Madalo Mukoka, Monde Muyoyeta, Elizabeth Nakabugo, Jerry Hella, Hung Van Nguyen, Van Anh Thi Nguyen, Sasiwimol Ubolyam, Berra Erkosar, Marcia Vermeulen, Chad M Centner, Sarah Nyangu, Nsala Sanjase, Andrea Cavallini, Aurélien Macé, Brian Shuma, Mohamed Sasamalo, Huong Thi Dinh, The Anh Ngo, Weerawat Manosuthi, Supunnee Jirajariyavej, Derek T Armstrong, Sergio Carmona, Tobias Broger, Apichaya Khlaiphuengsin, Aphicha Mahanontharit, Trang Thi Thu Pham, Hieu Thi Nguyen, Quang Van Pham, Nhung Viet Nguyen, Anchalee Avihingsanon, Andrew D Kerkhoff, Claudia M Denkinger, Klaus Reither, Lydia Nakiyingi, Prof Peter MacPherson, Prof Graeme Meintjes, Morten Ruhwald

**Affiliations:** a Department of Medicine, Faculty of Health Sciences, University of Cape Town, Cape Town, South Africa; b FIND, Geneva, Switzerland; c Public Health Group, Malawi-Liverpool-Wellcome Programme, Blantyre, Malawi; d Department of Pathology, Kamuzu University of Health Sciences, Blantyre, Malawi; e Centre for Infectious Diseases Research in Zambia, Lusaka, Zambia; f Infectious Diseases Institute, Makerere University, Kampala, Uganda; g Ifakara Health Institute, Dar es Salaam, Tanzania; h National Lung Hospital, Ha Noi, Viet Nam; i HIV-NAT, Thai Red Cross AIDS Research Centre, Bangkok, Thailand; j Center of Excellence in Tuberculosis, Faculty of Medicine, Chulalongkorn University, Bangkok, Thailand; k Wellcome Center for Infectious Diseases Research in Africa, Institute of Infectious Disease and Molecular Medicine, University of Cape Town, Cape Town, South Africa; l Division of Medical Microbiology, University of Cape Town, Cape Town, South Africa; m National Health Laboratory Service, Groote Schuur Hospital, Cape Town, South Africa; n Ifakara Health Institute, Dar es Salaam, Tanzania; o Viet Tiep Hospital, Hai Phong, Viet Nam; p Bamrasnaradura Infectious Diseases Institute, Nonthaburi, Thailand; q Taksin Hospital, Bangkok, Thailand; r Division of HIV, Infectious Diseases, and Global Medicine, Zuckerberg San Francisco General Hospital and Trauma Center, University of California San Francisco, San Francisco, CA, USA; s Division of Infectious Disease and Tropical Medicine, Heidelberg University Hospital, Heidelberg, Germany; t Faculty of Medicine, Heidelberg University, Heidelberg, Germany; u German Centre for infection Research (DZIF), partner site Heidelberg University Hospital, Heidelberg, Germany; v Swiss Tropical and Public Health Institute, Allschwil, Switzerland; w University of Basel, Basel, Switzerland; x School of Health and Wellbeing, University of Glasgow, Glasgow, UK; y Clinical Research Department, London School of Hygiene & Tropical Medicine, London, UK

## Abstract

**Background:**

Diagnostic delays for tuberculosis are common, with high resultant mortality. Urine-Xpert Ultra (Cepheid) could improve time to diagnosis of tuberculosis disease and rifampicin resistance. We previously reported on lot-to-lot variation of the Fujifilm SILVAMP TB LAM. In this prespecified secondary analysis of the same cohort, we aimed to determine the diagnostic yield and accuracy of Urine-Xpert Ultra for tuberculosis in people with HIV, compared with an extended microbiological reference standard (eMRS) and composite reference standard (CRS) and also compared with Determine TB LAM Ag (AlereLAM, Abbott).

**Methods:**

In this prospective, multicentre, diagnostic accuracy study, we recruited consecutive inpatients and outpatients (aged ≥18 years) with HIV from 13 hospitals and clinics in seven countries (Malawi, South Africa, Tanzania, Thailand, Uganda, Viet Nam, and Zambia). Patients with no isoniazid preventive therapy in the past 6 months and fewer than three doses of tuberculosis treatment in the past 60 days were included. Reference and index testing was performed in real time. The primary outcome of this secondary analysis was the diagnostic yield and accuracy of Urine-Xpert Ultra compared with the eMRS and CRS. Diagnostic accuracy was compared with AlereLAM and diagnostic yield was compared with both AlereLAM and Sputum-Xpert Ultra. This study was registered with ClinicalTrials.gov, NCT04089423, and is complete.

**Findings:**

Between Dec 13, 2019, and Aug 5, 2021, 3528 potentially eligible individuals were screened and 1731 were enrolled, of whom 1602 (92·5%) were classifiable by the eMRS (median age 40 years [IQR 33–48], 838 [52·3%] of 1602 were female, 764 [47·7%] were male, 937 [58·5%] were outpatients, 665 [41·5%] were inpatients, median CD4 count was 374 cells per μL [IQR 138–630], and 254 [15·9%] had microbiologically confirmed tuberculosis). Against eMRS as reference, sensitivities of Urine-Xpert Ultra and AlereLAM were 32·7% (95% CI 27·2–38·7) and 30·7% (25·4–36·6) and specificities were 98·0% (97·1–98·6) and 90·4% (88·7–91·8), respectively. Against CRS as reference, sensitivities of Urine-Xpert Ultra and AlereLAM were 21·1% (95% CI 17·6–25·1), and 30·5% (26·4–34·9), and specificities were 99·1% (98·3–99·6) and 95·1% (93·5–96·3), respectively. The combination of Sputum-Xpert Ultra with AlereLAM or Urine-Xpert Ultra diagnosed 202 (77·1%) and 204 (77·9%) of 262 eMRS-positive participants, respectively, in incompletely overlapping groups; combining all three tests diagnosed 214 (81·7%) of 262 eMRS-positive participants

**Interpretation:**

Urine-Xpert Ultra could offer promising clinical utility in addition to AlereLAM and Sputum-Xpert Ultra. In inpatient settings where both AlereLAM and Urine-Xpert Ultra are possible, both should be offered to support rapid diagnosis and treatment.

**Funding:**

Global Health Innovative Technology Fund, KfW Development Bank, Commonwealth of Australia represented by the Department of Foreign Affairs and Trade, and the Netherlands Enterprise Agency.

## Introduction

Tuberculosis is a leading infectious cause of death worldwide^1^ and among people with HIV it is the leading cause of death and hospitalisation.^2^, ^3^ People with HIV and tuberculosis often die before diagnosis or soon after treatment initiation, after diagnostic delays.^4^, ^5^, ^6^ People with HIV might also be unable to produce sputum that is commonly used for tuberculosis diagnosis due to severe illness, mental state alterations, or exclusively extrapulmonary disease, and reliance on sputum testing might lead to missed diagnoses.^7^ An urgent call has been made for rapid, non-sputum-based diagnostics of tuberculosis that can, ideally, be performed at point of care.^8^

Two randomised controlled trials have shown a survival benefit when a Determine TB LAM Ag assay (AlereLAM; Abbott, Chicago, IL, USA) was available for ill, hospitalised people with HIV. Therefore, WHO expanded their AlereLAM recommendations in 2019,^9^, ^10^, ^11^ such that for all hospitalised people with HIV who present with signs or symptoms of tuberculosis, signs of serious illness, or CD4 counts of less than 200 cells per μL, and outpatients with HIV who present with signs or symptoms of tuberculosis, signs of serious illness, or CD4 counts of less than 100 cells per μL are recommended to undergo an AlereLAM test. AlereLAM is performed on a urine sample and is the only commercially available, point-of-care tuberculosis test that can give a rapid result (ie, in under 30 min), although it cannot identify mycobacterial species or confirm drug susceptibility. Despite global recommendations and strong supportive evidence, AlereLAM is not routinely available in many high tuberculosis burden countries.^12^ Real-world data for the pragmatic use of AlereLAM are scarce but implementation is probably affected by various issues in clinical settings.^12^, ^13^, ^14^

**Research in context**

**Evidence before this study**

We searched PubMed on Aug 7, 2024, without date or language restrictions, for research articles that evaluated the diagnostic accuracy of Urine Xpert using the search terms (“tuberculosis” OR “TB”) AND (“urine” OR “urinary”) AND (“Xpert” OR “cepheid”). Our search identified 31 relevant studies, of which 23 assessed the MTB/RIF cartridge and eight assessed the Ultra cartridge. Cohorts included people living with or without HIV, or a combination, and adults and paediatric populations. When available, estimated sensitivities ranged from 0% to 100% with the MTB/RIF cartridge and from 14% to 41% with the Ultra cartridge. Reported specificities ranged from 54% to 100% with the MTB/RIF cartridge and from 98% to 99% with the Ultra cartridge. Diagnostic yield was rarely reported but ranged from 5% to 78% for the MTB/RIF cartridge and from 2·3% to 14% for the Ultra cartridge. To our knowledge, the largest study to investigate Ultra to date had 732 participants recruited from a single ambulatory centre in South Africa. One randomised controlled trial (the STAMP trial) assessed the effect of the addition of AlereLAM and Urine Xpert MTB/RIF testing in comparison with sputum Xpert MTB/RIF alone. In STAMP, against a microbiologically confirmed tuberculosis reference standard, the diagnostic yield of AlereLAM was 75% compared with a diagnostic yield of 35% for urine Xpert MTB/RIF. We did not identify any interventional trials that evaluated the clinical effect of Urine-Xpert Ultra in patient management. In a cost-effectiveness study based on the STAMP trial cohort, availability of concentrated urine Xpert MTB/RIF in addition to Sputum Xpert MTB/RIF and AlereLAM was deemed to be cost-effective.

**Added value of this study**

To our knowledge, this is the largest multicountry study to report on the diagnostic accuracy of Urine-Xpert Ultra in adults with HIV who are being investigated for presumptive tuberculosis. This study also has the strength of being performed in the era of Sputum-Xpert Ultra, and the ability to study the incremental benefit of Urine-Xpert Ultra in addition to Sputum-Xpert Ultra and AlereLAM. We found similar sensitivity and diagnostic yield between Urine-Xpert Ultra and AlereLAM, but a higher specificity and positive predictive value (PPV) with Urine-Xpert Ultra. Importantly, the numerically similar diagnostic yield included patients who were only diagnosed on one of Urine-Xpert Ultra, AlereLAM, or Sputum Xpert Ultra. The addition of either AlereLAM or Urine-Xpert Ultra to Sputum-Xpert Ultra was able to diagnose approximately 77% of microbiologically confirmed cases of tuberculosis. In post-hoc analyses, participants with Urine-Xpert Ultra positive results were also more likely to die within 10 weeks than were those with negative results, demonstrating the test's potential value in earlier diagnosis and initiation of tuberculosis treatment to reduce tuberculosis deaths.

**Implications of all the available evidence**

Although Urine-Xpert Ultra is not routinely performed, it has a similar sensitivity to AlereLAM and a greater specificity and PPV, is able to confirm *Mycobacterium tuberculosis* versus non-tuberculous mycobacteria, and can assess for rifampicin resistance. A limitation of Urine-Xpert Ultra compared with AlereLAM is higher cost and need for laboratory infrastructure. Considering resource implications, in inpatient settings where people living with HIV are at increased risk of early mortality, Urine-Xpert Ultra should be offered in addition to Sputum-Xpert Ultra and AlereLAM, particularly in those unable to produce sputum. This would facilitate rapid diagnosis and treatment initiation in severely ill people living with HIV who are being evaluated for tuberculosis, and at high mortality risk if appropriate tuberculosis treatment is delayed.

Urine samples can also be tested with the GeneXpert platform (Xpert; Cepheid, Sunnyvale, CA, USA) and, since 2017, transition has occurred to the Xpert MTB/RIF Ultra optimised cartridges.^15^ The Xpert platform is available in many high tuberculosis burden settings, and Urine-Xpert Ultra was included in a WHO policy brief^16^ in 2023 as an option for diagnosis of extrapulmonary tuberculosis in people with advanced HIV, but this test is seldom performed. A Cochrane review of Urine-Xpert Ultra has summarised the accuracy of Urine-Xpert Ultra for exclusively genitourinary tuberculosis, but not generally for people living with HIV being evaluated for tuberculosis.^17^ Xpert assays are performed in a laboratory, rather than at point of care, but can give confirmation of tuberculosis disease and can diagnose rifampicin resistance within 2 h of testing.

Recent promising evidence from studies evaluating the Fujifilm SILVAMP TB LAM (FujiLAM; Fujifilm, Tokyo, Japan) using frozen, biobanked urine^18^ led us to conduct a prospective multicentre diagnostic accuracy study of FujiLAM, the primary objective of which has been previously reported.^19^ We found lot-to-lot variability in FujiLAM diagnostic accuracy. In this analysis of secondary objectives, we aimed to determine the diagnostic yield and accuracy of Urine-Xpert Ultra and hypothesised that Urine-Xpert Ultra testing could improve the accuracy of current rapid tuberculosis diagnosis and detection of rifampicin resistance in people with HIV at high risk of mortality.

## Methods

### Study design and participants

In this prospective, multicentre, diagnostic accuracy study, participants were enrolled at 13 hospitals and clinics, ranging from primary care to referral centres, across seven high tuberculosis burden countries (Malawi, South Africa, Tanzania, Thailand, Uganda, Viet Nam, and Zambia). Participant enrolment procedures have been described previously.^19^ Briefly, screening and enrolment were offered to consecutive people with HIV who were admitted to hospital or attending outpatient clinics. Eligible individuals were aged 18 years or older and living with HIV (ie, self-reported or documented as HIV positive), willing to attend follow-up appointments, had received fewer than three doses of anti-tuberculosis treatment in the past 60 days, and no isoniazid preventive therapy during the past 6 months. In inpatient settings, enrolment was irrespective of tuberculosis symptoms, whereas in outpatient settings, at least one WHO-defined tuberculosis screening symptom (ie, cough, fever, night sweats, or weight loss) was required. Enrolment did not affect standard of care, but the research team did communicate results of study tests to treating clinicians that would have otherwise been available in that setting under standard care (appendix 1 p 4). Notably, sites in six study countries communicated AlereLAM results and sites in five countries communicated Urine-Xpert Ultra results to the treating clinicians and this reporting was done within 24 h of testing, allowing for rapid effect on treatment. We aimed to enrol participants soon after hospital admission (≤48 h) or ambulatory presentation (≤72 h).

All participants gave written informed consent and approvals were granted from Research Ethics Committees at each site. The study protocol is available in appendix 1 (pp 15–68). This study was registered with ClinicalTrials.gov, NCT04089423. This study is reported in accordance with the Standards for Reporting of Diagnostic Accuracy Study guidelines (appendix 1 p 6).

### Procedures

Blood and sputum samples were collected for reference standard testing and urine for index testing in accordance with the study protocol (appendix 1 pp 15–68). Baseline testing on day 1 (ie, the day of enrolment; blood, urine, and sputum) and day 2 (sputum and urine) was followed by further testing after 2–3 months (sputum only) in participants without microbiological confirmation of tuberculosis, who had not started on tuberculosis treatment, and in outpatients with unresolved tuberculosis symptoms (and irrespective of symptoms in inpatients). Urine and sputum samples were collected by clinically trained research staff, with sputum induction available before the COVID-19 pandemic reached their setting. All samples were collected, tested, and processed on-site (point-of-care tests) or at nearby accredited laboratories with standardised protocols.

The protocol defined extended microbiological reference standard (eMRS)-positive participants as those who had any sputum-based, blood-based, or other clinically indicated test positive for *M tuberculosis* and eMRS-negative participants were defined as those in whom these tests were not positive, including at least one negative sputum culture (the tests used and reference definitions are provided in appendix 1 [p 5]). Participants who were positive by composite reference standard (CRS) were defined as eMRS-positive participants, or those in whom tuberculosis treatment was started and who had a positive response to treatment, or both. CRS-negative participants were those in whom none of the eMRS tests were positive, no tuberculosis treatment had been prescribed, and who were asymptomatic at the 2–3-month follow-up visit. Two reference standards (ie, eMRS and CRS) were used, per good practice for non-sputum tuberculosis diagnostic studies.^20^ Participants without a valid index test or for whom reference standard assessment was neither positive nor negative were deemed to be unclassifiable and not included in the analyses of sensitivity, specificity, positive predictive value (PPV), or negative predictive value (NPV). The analysis of diagnostic yield was conducted in comparison with the eMRS but did not require a valid index test result. Diagnostic yield was calculated as the proportion participants who were test (or tests) positive divided by the total number of non-index tests that confirmed tuberculosis. Urine-Xpert Ultra and AlereLAM results did not contribute to reference standard classification or the diagnostic yield denominator. Any reference to microbiologically confirmed tuberculosis was based on Xpert (other than on urine alone) or mycobacterial cultures being positive for *Mycobacterium tuberculosis* (ie, eMRS-positive).

Reference standard testing included Sputum-Xpert Ultra and mycobacterial culture on blood and sputum (detailed methods are in appendix 1 [p 14]). Clinically indicated mycobacterial tests that were performed by the routine treating clinicians were also recorded for reference standard assessment. Assessors of the reference standard were masked to the index test results. For the purposes of subgroup analyses, CD4 cell counts were collected at enrolment.

AlereLAM was done according to manufacturer's instructions on the same day as sample collection. Briefly, 60 μL urine was applied to the sample pad. After 25 min, test strips were read using the reference card to confirm the grade 1 threshold for positivity. Invalid AlereLAM results (ie, absent control line) were repeated once from the same urine sample. For Urine-Xpert Ultra testing, urine was transported at 2–8°C to the laboratories mentioned earlier for same day testing. Up to 30 mL of urine was centrifuged at 3000 g for 15 min, then the pellet was resuspended in 0·75 mL phosphate buffered saline and 1·5 mL of Xpert reagent buffer. Any detectable *M tuberculosis* DNA in urine was deemed to be a positive result, including trace *M tuberculosis* results. Urine-Xpert Ultra tests for which the initial result was invalid could not be repeated. Assessors of the index tests were masked to the eMRS.

For those participants that did not attend the study visit at 2–3 months, data on vital status outcomes was supplemented via electronic health records.

### Outcomes

The primary outcome of this analysis of secondary objectives was the diagnostic yield and accuracy of Urine-Xpert Ultra compared with the eMRS and CRS, and in comparison with AlereLAM. Diagnostic yield was studied in comparison with AlereLAM or Sputum-Ultra Xpert, or both.

### Statistical analysis

To investigate diagnostic accuracy, we calculated point estimates and Wilson's 95% CIs for the sensitivity, specificity, PPV, NPV, and diagnostic yield—for Urine-Xpert Ultra and AlereLAM, separately, and in combination—using the eMRS and CRS as the reference standards in separate analyses. In prespecified subgroup analyses, diagnostic accuracy was estimated by CD4 strata (≤200 cells per μL *vs* >200 cells per μL) and setting (outpatient *vs* inpatient). Diagnostic yield was estimated, with the eMRS as reference, overall and in the same subgroups. In a post-hoc analysis, we investigated associations between Urine-Xpert Ultra and 10-week all-cause mortality initially in a complete-case analysis, which was limited to participants with complete data for CD4 count, sex, antiretroviral therapy (ART) status, index tests, and with a documented vital status outcome at 10 weeks (ie, known to be alive or dead, and excluding those lost to follow-up). We calculated the difference in 10-week mortality between participants with and without confirmed tuberculosis using the χ^2^ test, and then a univariable analysis to assess 10-week mortality by test result. To estimate odds ratios (ORs), 95% CIs, and conditional average effects of Urine-Xpert Ultra positivity on 10-week mortality, random-effects multivariable logistic regression models were constructed, adjusting a priori for prespecified variables based on a directed acyclic graph,^21^ and including natural cubic spline terms with three knots for age and CD4 count. We included a random intercept term for study country. In prespecified sensitivity analysis, to account for missing data, we fitted models using 25 multiply imputed datasets (using predictive mean matching algorithms, and with outcome included as a predictor of missingness) and summarised the data using Rubin's rules.^22^, ^23^ Diagnostic yield was also assessed post-hoc for the subgroup that died due to any cause versus those that were alive at 10 weeks.

In a post-hoc analysis, we assessed 10-week mortality in participants with positive mycobacterial blood cultures (ie, tuberculosis bloodstream infections) and analysed diagnostic yields of each test in this population. Additionally, to assess concordance between rifampicin susceptibility results on urine and other tests, we compared rifampicin results from Urine-Xpert Ultra and study Sputum-Xpert Ultra results in a post-hoc analysis.

Sample size calculations were based on the expected diagnostic accuracy of Fujifilm SILVAMP TB LAM (FujiLAM), as per our protocol's primary objectives, the results of which are reported elsewhere.^19^ With an assumed 60% overall sensitivity for the index test, a sample size of 223 individuals with confirmed tuberculosis was necessary to estimate sensitivity with 80% power, an alpha error of 0·05, and with a precision of 9% for the primary analysis. No post-hoc sample size calculations were done for the secondary analysis, since this is discouraged.

The analysis was conducted in R (version 4.0.3).

### Role of the funding source

The funders had no role in study design, data collection, data analysis, data interpretation, or manuscript writing.

## Results

**Figure 1:**
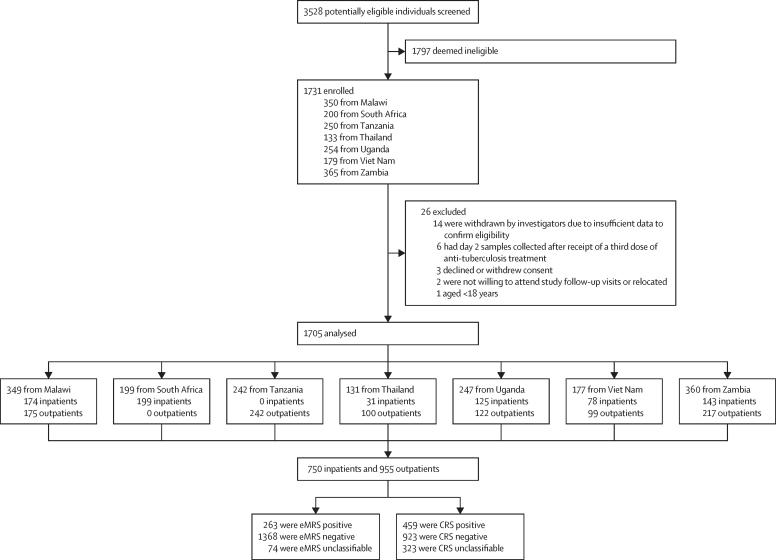
Study flow diagram 1797 were deemed ineligible per protocol for the following reasons: due to already having had three doses antituberculosis treatment in the past 60 days, having received isoniazid prophylaxis therapy in the past 6 months, being outpatients who had no symptoms of tuberculosis, or being unwilling to attend trial follow-up; exact numbers were not captured. CRS=composite reference standard. eMRS=extended microbiological reference standard.

Between Dec 13, 2019, and Aug 5, 2021, 3528 potentially eligible individuals were screened and 1731 were enrolled (figure 1). After 26 exclusions, 750 inpatients and 955 outpatients with HIV were included. Of 1705 participants, 1598 (93·7%) could produce sputum, 1703 (99·9%) produced urine, and 1688 (96·5%) had mycobacterial blood cultures. Overall, 263 (15·4%) of 1705 participants had Xpert-confirmed or culture-confirmed *M tuberculosis* from a pulmonary or extra-pulmonary sample (excluding those who were positive by Urine-Xpert Ultra alone). 74 (4·3%) of 1705 participants were unclassifiable by eMRS and 103 (6·0%) were deemed unclassifiable by eMRS or index testing. 323 (18·9%) were unclassifiable by CRS and 346 (20·3%) were deemed unclassifiable by CRS or index testing. 21 (1·2%) of 1705 participants did not have a Urine-Xpert Ultra result (eight [0·5%] had an invalid result and 13 [0·8%] had insufficient volume or were not tested) and 13 (0·8%) did not have an AlereLAM result (five [0·3%] had an invalid result and eight [0·5%] had insufficient volume or were not tested).

Tuberculosis prevalence among people with HIV differed by site (appendix 1 p 8). Participants had a median of four reference standard tuberculosis tests (IQR 4–6; range 0–14) and a median of four tuberculosis tests were done in both eMRS-positive (IQR 4–4) and eMRS-negative (4–7) participants (appendix 1 p 8). A similar distribution was also seen between Urine-Xpert Ultra-positive and AlereLAM-positive groups (median of four tests [IQR 4–4 for both]).

Participants classifiable in the eMRS accuracy analysis (n=1602) had a median age of 40 years (IQR 33–48), and 838 (52·3%) were female and 764 (47·7%) were male (table; no data were collected on race or ethnicity). Microbiologically confirmed tuberculosis was diagnosed in 254 (15·9%) of 1602 participants. An additional 237 (17·6%) of 1348 eMRS-negative participants were started on tuberculosis treatment by their treating clinician. Overall, 665 (41·5%) of 1602 classifiable participants were inpatients (appendix 1 pp 9, 13).

**Table:** Demographic and clinical characteristics of classifiable participants at enrolment, with stratification by eMRS status

		All participants (N=1602)*	**eMRS-positive participants (n=254)**	**eMRS-negative participants (n=1348)**	**p value**
Age, years		40 (33–48)	39 (32–46)	41 (33–48)	0·075
Sex					
	Female	838 (52·3%)	114 (44·9%)	724 (53·7%)	0·0098
	Male	764 (47·7%)	140 (55·1%)	624 (46·3%)	..
ART status at enrolment					
	Treatment interruption	88 (5·5%)	24 (9·4%)	64 (4·7%)	0·0026
	Currently on ART	1246 (77·8%)	168 (66·1%)	1078 (80·0%)	<0·0001
	ART naive	250 (15·6%)	59 (23·2%)	191 (14·2%)	0·0003
	Unknown	18 (1·1%)	3 (1·2%)	15 (1·1%)	..
CD4 count, cells per μL					
	Median	374 (138–630)	176 (51–452)	406 (171–657)	<0·0001
	≤200	504 (31·5%)	128 (50·4%)	376 (27·9%)	<0·0001
	>200	1082 (67·5%)	121 (47·6%)	961 (71·3%)	<0·0001
	Unknown	16 (1·0%)	5 (2·0%)	11 (0·8%)	..
Self-reported history of tuberculosis		421 (26·3%)	77 (30·3%)	344 (25·5%)	0·11
Seriously ill†		698 (43·6%)	148 (58·3%)	550 (40·8%)	<0·0001
WHO symptom screen positive‡		1491 (93·1%)	248 (97·6%)	1243 (92·2%)	0·0017
Setting					
	Inpatient	665 (41·5%)	130 (51·2%)	535 (39·7%)	0·0007
	Outpatient	937 (58·5%)	124 (48·8%)	813 (60·3%)	..
Vital status at 10 weeks					
	Alive	1379 (86·1%)	222 (87·4%)	1157 (85·8%)	0·51
	Died	115 (7·2%)	24 (9·4%)	91 (6·8%)	0·13
	Lost to follow-up	108 (6·7%)	8 (3·1%)	100 (7·4%)	0·0128

Data are median (IQR) or n (%). p values are for comparison between eMRS-positive and eMRS-negative participants, using the χ^2^ or Wilcoxon rank-sum tests as appropriate. ART=antiretroviral therapy. eMRS=extended microbiological reference standard.

* 103 (6·0%) of 1705 participants were deemed unclassifiable by eMRS or index testing (ie, Urine-Xpert Ultra and AlereLAM). eMRS positive participants had any sputum-based, blood-based, or other clinically indicated test positive for *Mycobacterium tuberculosis*; whereas the eMRS negative participants were those in whom these tests were not positive, including at least one negative sputum culture (appendix 1 p 5).

† Criteria based on WHO-defined seriously ill criteria—ie, any of: BMI of <18·5 kg/m^2^, respiratory rate of >30 breaths per min, or systolic blood pressure <90 mm Hg, heart rate >120 beats per min, or inability to walk unaided.

‡ At least one for the following four symptoms present: cough, weight loss, night sweats, or fever.

**Figure 2:**
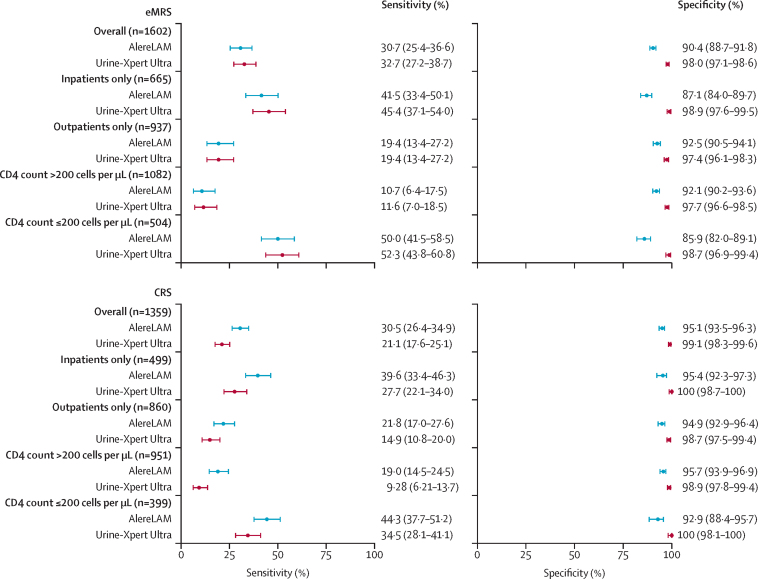
Sensitivity and specificity of AlereLAM and Urine-Xpert Ultra in the overall cohort versus the eMRS and CRS, and in subgroups defined by CD4 count and setting Error bars are Wilson's 95% CIs. Numbers of true positives, false positives, true negatives, and false negatives for each subgroup are in appendix 1 (p 11). CRS=composite reference standard. eMRS=extended microbiological reference standard.

Using eMRS as the reference standard, sensitivities of AlereLAM and Urine-Xpert Ultra were 30·7% (95% CI 25·4–36·6) and 32·7% (27·2–38·7), respectively (figure 2; appendix 1 p 11). The specificities against eMRS of AlereLAM and Urine-Xpert Ultra were 90·4% (95% CI 88·7–91·8) and 98·0% (97·1–98·6), respectively. In all eMRS subgroups defined by CD4 cell count and setting, AlereLAM and Urine-Xpert Ultra had overlapping 95% CIs for sensitivity, with both index tests having sensitivity gains in inpatients and the group with low CD4 count (figure 2; appendix 1 p 11). Across all eMRS subgroups, the specificity of Urine-Xpert Ultra was always above 97% and was more specific than AlereLAM, with non-overlapping 95% CIs.

Against the eMRS, which identified a tuberculosis prevalence of 15·9% overall, the PPV of AlereLAM was 37·5% (95% CI 31·2–44·2) and the NPV was 87·4% (85·5–89·0; appendix 1 p 11). In the same group, the PPV of Urine-Xpert Ultra was 75·5% (66·6–82·5) and the NPV was 88·5% (86·8–90·1). In all eMRS subgroups, the PPV of Urine-Xpert Ultra was approximately double that of AlereLAM, but the NPVs were similar (appendix 1 p 11).

446 (32·8%) of 1359 participants were positive on the CRS. Using CRS as the reference standard, the sensitivities of AlereLAM and Urine-Xpert Ultra were 30·5% (95% CI 26·4–34·9) and 21·1% (17·6–25·1), respectively (figure 2; appendix 1 p 11). The specificities against CRS for AlereLAM and Urine-Xpert Ultra were 95·1% (95% CI 93·5–96·3) and 99·1% (98·3–99·6), respectively. Sensitivities and specificities in CRS subgroups defined by CD4 count and setting are shown in figure 2 and appendix 1 (p 11). In all CRS subgroups, AlereLAM and Urine-Xpert Ultra had overlapping 95% CIs for sensitivity, and Urine-Xpert Ultra was more specific than AlereLAM, with non-overlapping 95% CIs. Clinical details of the eight participants deemed to have false-positive Urine-Xpert Ultra results against the CRS are in appendix 1 (p 10). They were all outpatients with CD4 counts of 341 cells per μL or higher, six of eight had chest x-rays that were described by investigators as showing either “tuberculosis likely” or “pneumonia”, and all were documented as having symptom improvement without tuberculosis treatment at the 2–3 month study visit.

**Figure 3:**
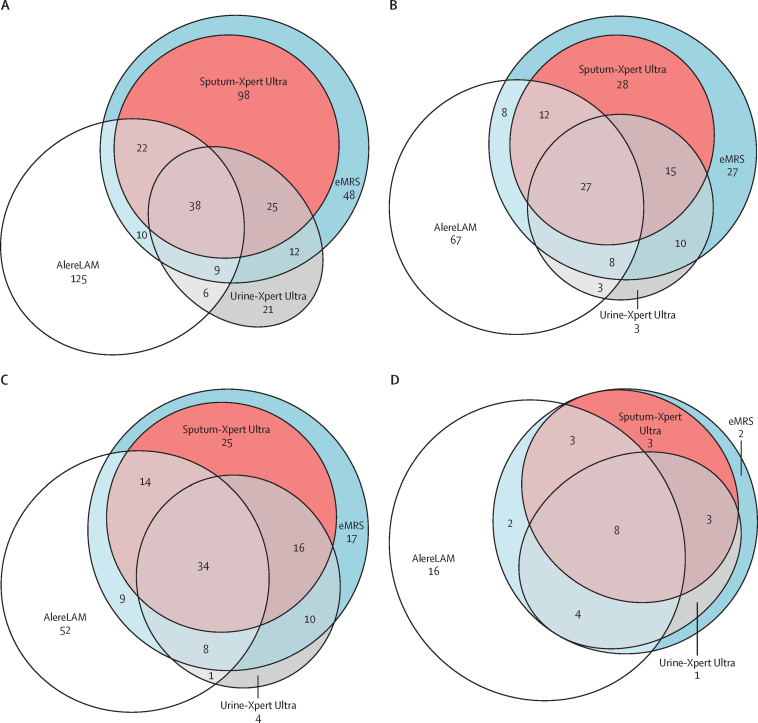
Diagnostic yield per rapid test in all participants (A), only inpatients (B), those with CD4 counts of ≤200 cells per μL (C), and among those who died within 10 weeks of enrolment (D) compared with the eMRS-positive group eMRS=extended microbiological reference standard.

AlereLAM diagnosed 79 (30·2%) of 262 eMRS-positive participants and Urine-Xpert Ultra diagnosed 84 (32·1%) eMRS-positive participants (figure 3A). The combination of Sputum-Xpert Ultra and AlereLAM diagnosed 202 (77·1%) of 262 eMRS-positive participants and Sputum-Xpert Ultra with Urine-Xpert Ultra diagnosed 204 (77·9%) of 262 participants—including some patients who were only diagnosed by one of these tests. 79 (30·2%) of 262 eMRS-positive participants had tuberculosis diagnosed via tests other than Sputum-Xpert Ultra. Combining Sputum-Xpert Ultra, Urine-Xpert Ultra, and AlereLAM diagnosed 214 (81·7%) of 262 eMRS-positive participants. An additional 131 participants were AlereLAM positive, and another 27 were Urine-Xpert Ultra positive, but negative on the eMRS (appendix 1 p 14).

In eMRS-classifiable inpatients, 643 (94·1%) of 683 were able to produce a sputum sample and 682 (99·9%) were able to produce a urine sample. When limiting the analysis to only eMRS-positive inpatients, the diagnostic yield of each test was 55 (40·7%) of 135 for AlereLAM, 60 (44·4%) of 135 for Urine-Xpert Ultra, and 82 (60·7%) of 135 for Sputum-Xpert Ultra (figure 3B). Combining all three rapid tests, the diagnostic yield was 80·0% (108 of 135). Diagnostic yields for the eMRS-classifiable outpatient population are in appendix 1 (p 13).

In the 515 participants classifiable by the eMRS who had CD4 counts of 200 cells per μL or lower, 491 (95·3%) could produce sputum and 514 (99·8%) could produce urine. When limiting the analysis to eMRS-positive participants with CD4 counts of 200 cells per μL or lower, the diagnostic yield of each test was 65 (48·9%) of 133 for AlereLAM, 68 (51·1%) of 133 for Urine-Xpert Ultra, and 89 (66·9%) of 133 for Sputum-Xpert Ultra (figure 3C). Combining all three rapid tests, the diagnostic yield was 87·2% (116 of 133). Diagnostic yields for the eMRS-classifiable population with CD4 counts of more than 200 cells per μL are in appendix 1 (p 13).

26 (9**·**9%) of 263 participants with microbiologically confirmed tuberculosis were known to have died within 10 weeks, of whom 16 (61·5%) were positive on Urine-Xpert Ultra, 17 (65·4%) on Sputum-Xpert Ultra, and 17 (65·4%) on AlereLAM (figure 3D). By combining all three tests, the diagnostic yield was 92·3% (24 of 26). Diagnostic yields when limiting to those who were alive at 10 weeks are in appendix 1 (p 13).

**Figure 4:**
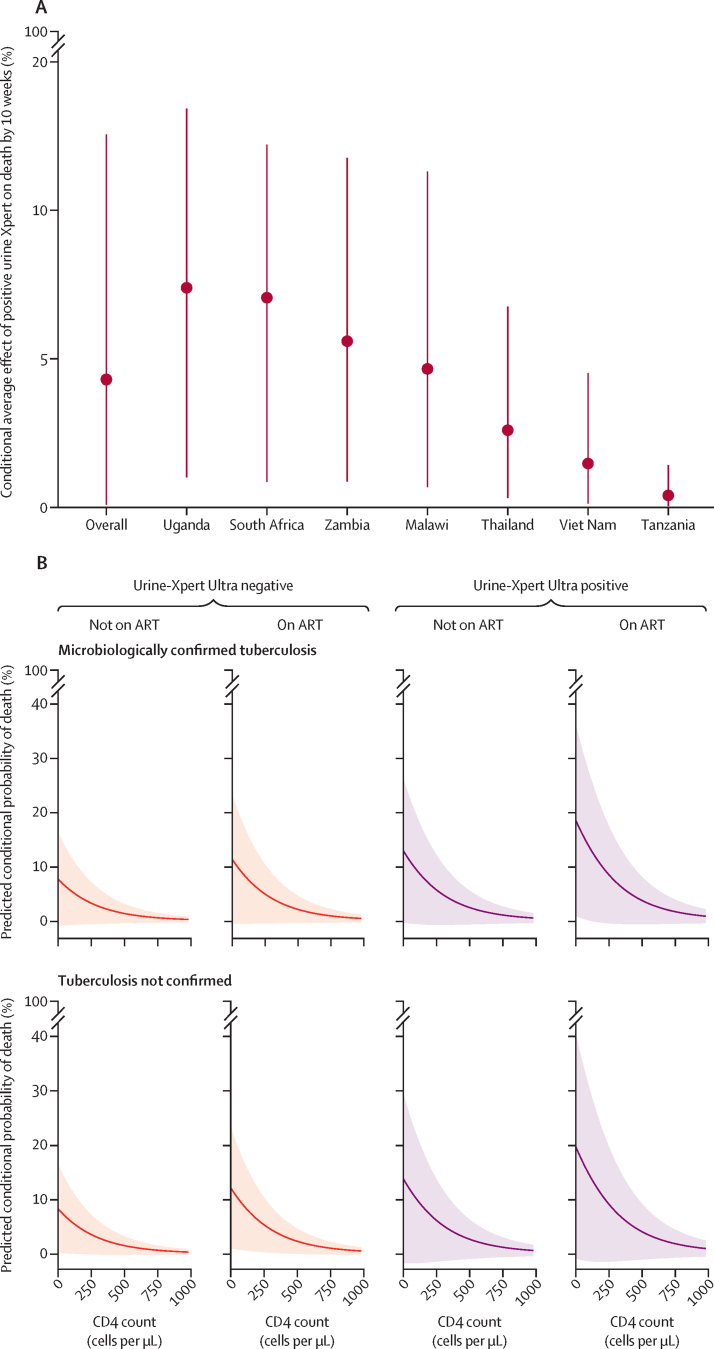

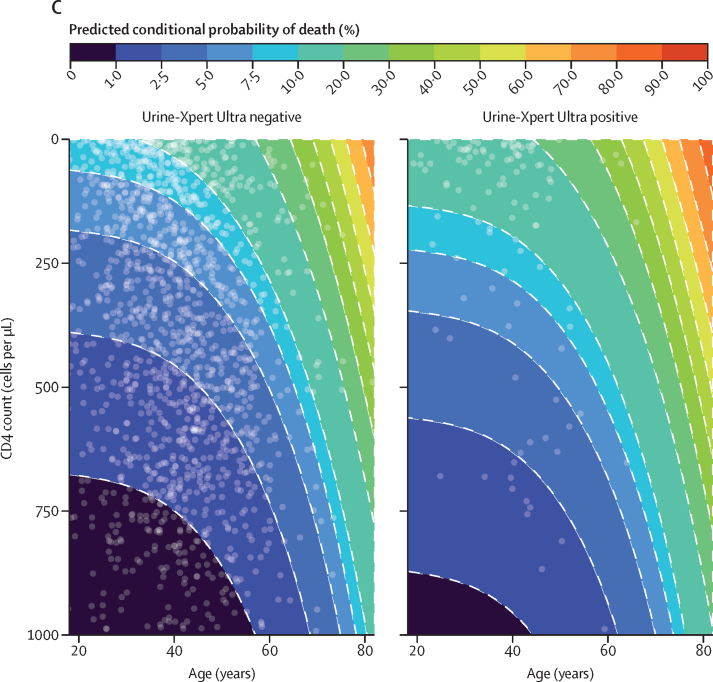
Urine-Xpert Ultra positivity and mortality (A) Overall and country-specific conditional average effect of positive urine Xpert Ultra on death, determined with a multilevel logistic regression model. (B) Predicted probability of death by CD4 count, conditional on antiretroviral status and tuberculosis extended microbiological reference standard results. (C) Predicted conditional probability of death by age and CD4 count by Urine-Xpert Ultra results; white dots are individual participants and colour segments are model predictions. ART=antiretroviral therapy.

For the complete-case analysis 1470 participants were included, of whom 112 (7·6%) had died within 10 weeks. 24 (10·1%) of 238 participants with microbiologically confirmed tuberculosis died compared with 88 (7·1%) of 1232 without confirmed tuberculosis (χ^2^ p=0·15). In univariable analysis, participants with positive Urine-Xpert Ultra results were significantly more likely to have died by 10 weeks than were those with negative Urine-Xpert Ultra results (16·5% [95% CI 9·5–23·5; 18 of 109] *vs* 7·8% [6·4–9·2; 111 of 1424]; average effect: 8·7% [95% CI 1·6 to 15·8]; OR 2·3 [95% CI 1·3 to 3·9]). In the random-effects multivariable logistic regression model, for participants with microbiologically confirmed tuberculosis who were on ART, with other variables held at their mean (ie, CD4 count and age), participants with positive Urine-Xpert Ultra results remained at higher risk of death by 10 weeks than those with negative results (adjusted OR 2·1 [95% CI 0·9–4·8]), with a conditional average effect on death by 10 weeks of 5·8% (95% CI 0·1–6·5). A similar trend was seen between AlereLAM and mortality (appendix 1 p 14). The conditional average effect of positive Urine-Xpert Ultra status on death by 10 weeks varied considerably by country, ranging from 0·6% (95% CI 0·1–2·1) in Tanzania (where only outpatients were enrolled), to 10·0% (1·5–17·7) in Uganda (where both inpatients and outpatients where enrolled; figure 4A). Additionally, we found that older age, lower CD4 cell count, and taking ART were associated with increased conditional average effects of Urine-Xpert Ultra positivity on death by 10 weeks (figure 4B, C). Sensitivity analysis by multiple imputation had minimal effects on the effect estimates (conditional average effect of Urine-Xpert Ultra-positivity on death: 6·0% [95% CI 0·1–16·7]).

In exploratory post-hoc analysis, seven (25·0%) of 28 participants with tuberculosis bloodstream infections died within 10 weeks. Among the 28 participants with tuberculosis bloodstream infections, 22 (78·6%) were positive on Urine-Xpert Ultra, 14 (50·0%) on Sputum-Xpert Ultra, and 17 (60·7%) on AlereLAM. 25 (89·3%) of 28 participants were diagnosed with at least one of these tests. Two (0·1%) of 1705 participants had non-tuberculous bloodstream infections, one of whom had a positive AlereLAM test, with all other tuberculosis tests being negative; Urine-Xpert Ultra was negative for both participants.

238 (14**·**0%) of 1705 participants had *M tuberculosis* detected on Sputum-Xpert Ultra or Urine-Xpert Ultra, and 12 (5·0%) of 238 had detected rifampicin resistance (appendix 1 p 12). In this same group, the prevalence of trace readings (ie, where rifampicin susceptibility is unknown) on Sputum-Xpert Ultra and Urine-Xpert Ultra was 57 (31·1%) of 183 and 35 (29·7%) of 118, respectively, and 163 (68·5%) of 238 had a confirmed sensitive or resistant result on either sample. No discrepancy in rifampicin sensitivity results were seen between Urine-Xpert Ultra and Sputum-Xpert Ultra tests.

## Discussion

To our knowledge, this multinational study in inpatients and outpatients with HIV is the largest to date to report on the diagnostic accuracy of Urine-Xpert Ultra. Urine-Xpert Ultra had similar sensitivity and diagnostic yield to AlereLAM, but significantly improved specificity and PPV. The combination of Urine-Xpert Ultra with AlereLAM and Sputum-Xpert Ultra rapidly diagnosed tuberculosis in 81·7% of participants overall or 87·2% of participants with CD4 counts of 200 cells per μL or lower. Furthermore, in post-hoc analyses, we found that Urine-Xpert Ultra could confirm *M tuberculosis* over non-tuberculosis mycobacteria, and accurately identified participants with rifampicin resistance.

We found Urine-Xpert Ultra positivity to be associated with 10-week mortality. A similar association between AlereLAM and mortality has been reported elsewhere^24^ and was seen again in this cohort. Randomised trials have shown survival benefit in ill, hospitalised patients when AlereLAM is available (in the era of Xpert MTB/RIF),^9^, ^10^ but no trials to our knowledge have assessed for survival benefit with Urine-Xpert Ultra. Urine-Xpert Ultra had the highest diagnostic yield in participants with tuberculosis bloodstream infection (22 [78·6%] of 28), a group known to be at risk of early mortality.^25^

In our study, we found AlereLAM to have lower sensitivity but similar specificity to that reported in a previous Cochrane review.^26^ Differing sensitivity might be driven by participants’ immune status, with most studies included in the Cochrane review reporting a median CD4 count of less than 250 cells per μL, compared with a median of 374 cells per μL in our classifiable population. Although the specificity of AlereLAM in our study is within the confidence bounds of the estimate in the Cochrane review, studies with more rigorous reference standards, such as the current study, have generally shown higher specificity.^26^

A key potential advantage of Urine-Xpert Ultra is increased confidence in positive results. In our cohort, Urine-Xpert Ultra had approximately double the PPV of AlereLAM against the eMRS. Urine-Xpert Ultra could reduce false positive diagnoses of tuberculosis in severely unwell people living with HIV. The potential reasons for false-positive AlereLAM results are incompletely understood but could include cross reactivity with environmental mycobacteria,^27^ so-called over-reading of AlereLAM tests in relation to the reference card, and might currently be influenced by epidemiology and the connected pre-test probability relating to the prevalence of various opportunistic infections.^28^

Specificity of both index tests against the CRS needs to be considered within the context that some sites communicated index test results to the treating clinicians in real time, which might have influenced tuberculosis treatment decisions. Notably, the specificity of Urine-Xpert Ultra was still higher than AlereLAM in all eMRS and CRS subgroups. By adding Urine-Xpert Ultra to the currently recommended AlereLAM and Sputum-Xpert Ultra algorithms, diagnostic yield for rapid tuberculosis diagnosis would increase without compromising specificity.

Although our study included both inpatients and outpatients, we found a median CD4 count of 374 cells per μL and a lower-than-expected mortality of 7·2% at 10 weeks, which is still unacceptably high. Hence, our cohort comprised individuals with less advanced HIV than in previous diagnostic studies in this field, in which participants had greater difficulty producing sputum or had more extrapulmonary or disseminated disease.^9^, ^10^, ^29^ Our cohort reflects contemporary HIV inpatient and outpatient populations, where ART coverage is high, and emphasises that sputum testing should still be done when possible. In our cohort, 93·7% of participants were able to produce at least one sputum sample and 99·9% produced a urine sample. A previous large meta-analysis showed that only 82% of people with HIV could provide sputum within 2 days whereas 98% could provide urine,^7^ suggesting that urine-based tests would be more useful in patients unable to produce sputum.

A 2019 cost-effectiveness study showed that using both Urine Xpert MTB/RIF and AlereLAM in addition to Sputum MTB/RIF was cost-effective, in both low-income and upper-middle-income settings.^30^ Furthermore, the cost of Ultra cartridges has decreased to US$7·97 for low-income and middle-income countries.^31^ Hence, updated cost-effectiveness analyses should be completed using our results for the Ultra cartridges and the pricing decrease.

This study has important limitations. We do not have rifampicin resistance data from all culture samples and so we could only inform rifampicin resistance analyses with study Xpert Ultra results. Additionally, not all sites were able to capture all clinically indicated non-study tuberculosis tests due to some sites not having electronic laboratory reporting systems; more advanced sampling and diagnostics (eg, a culture of pleural fluid or a biopsy of a lymph node that requires ultrasound guidance) also varied by site. To advance our understanding of Urine-Xpert Ultra, we also included post-hoc analyses along with our analysis of secondary objectives and note that sample size calculations were based on our protocol's primary objective. Finally, index test results were communicated to treating clinicians at some sites, which might have effected the reference standard allocations for the CRS, but not the eMRS. Treatment in these participants might have also positively affected patient outcomes—namely, survival. Whether these results were or were not communicated, participants still would have had systematic testing for tuberculosis on all microbiological reference standard tests. The strengths of the study are the large sample size, the use of two reference standards, and the multicounty design. Furthermore, this study went beyond diagnostic accuracy and assessed prognostic value, although this was done post hoc. Although data on Urine-Xpert Ultra also provides promise for other nucleic acid amplification test platforms in urine samples, these would need to be independently assessed.

In this large, multinational study of Urine-Xpert Ultra, we found that Urine-Xpert Ultra probably provides additional benefit over AlereLAM because it has similar sensitivity, but improved specificity and PPV. Considering resource implications, in inpatient settings where people living with HIV are at higher risk of early mortality, Urine-Xpert Ultra should be offered in addition to Sputum-Xpert Ultra and AlereLAM, particularly in those who are unable to produce sputum. This addition would facilitate rapid diagnosis and treatment initiation in severely ill people with HIV who are being evaluated for tuberculosis, and who are at high mortality risk if appropriate tuberculosis treatment is delayed.

### Contributors

### Equitable partnership declaration

### Data sharing

The study protocol is available in appendix 1. De-identified participant data can be shared upon reasonable request by contacting the corresponding author and would always require a data sharing agreement.

## Declaration of interests

RS, BE, CMD, and MR are or were employed by FIND, the global alliance for diagnostics, at the time of the study; FIND is a not-for-profit foundation that supports the evaluation of publicly prioritised tuberculosis assays and the implementation of WHO-approved (guidance and prequalification) assays using donor grants; FIND has product evaluation agreements with several private sector companies that design diagnostics for tuberculosis and other diseases; these agreements strictly define FIND's independence and neutrality with regard to these private sector companies. CMD reports funding from the US National Institutes of Health for the R2D2 project (1U01AI152087-01). All other authors declare no competing interests.

## Supplementary Materials

Supplementary appendix 1

Equitable Partnership Declaration
