# Supplementary material for: Urine-Xpert Ultra for the diagnosis of tuberculosis in people living with HIV: a prospective, multicentre, diagnostic accuracy study
Source: Lancet Glob Health. 2024 Nov 20;12(12):e2024–2034. doi: 10.1016/S2214-109X(24)00357-7 (PMC11584317; doi:10.1016/S2214-109X(24)00357-7)
Supplement: Supplementary appendix 1 [file mmc1.pdf]

THE LANCET
Global Health

Supplementary appendix 1

This appendix formed part of the original submission and has been peer reviewed. We post it as supplied by the authors.

Supplement to: Sossen B, Székely R, Mukoka M, et al. Urine-Xpert Ultra for the diagnosis of tuberculosis in people living with HIV: a prospective, multicentre, diagnostic accuracy study. *Lancet Glob Health* 2024; **12:** e2024–34.

Supplementary File for

Urine Xpert Ultra for the diagnosis of tuberculosis in people living with HIV: A prospective, multicentre diagnostic accuracy study

Full author list including FujiLAM Study Consortium:

1. Bianca Sossen (MBChB): Department of Medicine, Faculty of Health Sciences, University of Cape Town, Cape Town, South Africa
2. Rita Székely (PhD): FIND, Geneva, Switzerland
3. Madalo Mukoka (MSc): Public Health Group, Malawi-Liverpool-Wellcome Programme, Blantyre, Malawi & Department of Pathology, Kamuzu University of Health Sciences, Blantyre, Malawi
4. Monde Muyoyeta (PhD): Centre for Infectious Diseases research in Zambia, Lusaka, Zambia
5. Elizabeth Nakabugo (BSc): Infectious Diseases Institute, Makerere University, Kampala, Uganda
6. Jerry Hella (PhD): Ifakara Health Institute, Dar es Salaam, Tanzania
7. Hung Van Nguyen (PhD): National Lung Hospital, Ha Noi, Viet Nam
8. Van Anh Thi Nguyen (PhD): FIND, Hanoi, Viet Nam
9. Sasiwimol Ubolyam (PhD): HIV-NAT, Thai Red Cross AIDS Research Centre and Center of Excellence in Tuberculosis, Faculty of Medicine, Chulalongkorn University, Bangkok, Thailand
10. Berra Erkosar (PhD): FIND, Geneva, Switzerland
11. Marcia Vermeulen (MBChB): Wellcome Center for Infectious Diseases Research in Africa, Institute of Infectious Disease and Molecular Medicine, University of Cape Town, Cape Town, South Africa
12. Chad M Centner (MSc): Division of Medical Microbiology, University of Cape Town and National Health Laboratory Service, Groote Schuur Hospital, Cape Town, South Africa
13. Sarah Nyangu (MBChB): Centre for Infectious Diseases research in Zambia, Lusaka, Zambia
14. Nsala Sanjase (MBChB): Centre for Infectious Diseases research in Zambia, Lusaka, Zambia
15. Andrea Cavallini: FIND, Geneva, Switzerland
16. Aurélien Macé (PhD): FIND, Geneva, Switzerland
17. Brian Shuma (MPH): Centre for Infectious Diseases research in Zambia, Lusaka, Zambia

18. Mohamed Sasamalo (MSc): Ifakara Health Institute, Dar es Salaam, Tanzania
19. Huong Thi Dinh (MSc): National Lung Hospital, Ha Noi, Viet Nam
20. The Anh Ngo (PhD): Viet Tiep Hospital, Hai Phong, Viet Nam
21. Weerawat Manosuthi (MD): Bamrasnaradura Infectious Diseases Institute, Nonthaburi, Thailand
22. Supunnee Jirajariyavej (MD): Taksin Hospital Bangkok, Thailand
23. Derek T Armstrong (MHS): FIND, Geneva, Switzerland
24. Sergio Carmona: FIND, Geneva, Switzerland
25. Tobias Broger (MSc): FIND, Geneva Switzerland and Division of Infectious Disease and Tropical Medicine, Heidelberg University Hospital and Faculty of Medicine, Heidelberg University, Heidelberg, Germany
26. Apichaya Khlaiphuengsin: HIV-NAT, Thai Red Cross AIDS Research Centre and Center of Excellence in Tuberculosis, Faculty of Medicine, Chulalongkorn University, Bangkok, Thailand
27. Aphicha Mahanontharit: HIV-NAT, Thai Red Cross AIDS Research Centre and Center of Excellence in Tuberculosis, Faculty of Medicine, Chulalongkorn University, Bangkok, Thailand
28. Trang Thi Thu Pham (DM): Hai Phong University of Medicine and Pharmacy, Hai Phong, Viet Nam
29. Hieu Thi Nguyen (MMed): Viet Tiep Hospital, Hai Phong, Viet Nam
30. Quang Van Pham (MBBS): Hai Phong Lung Hospital, Hai Phong, Viet Nam
31. Nhung Viet Nguyen (PhD): National Lung Hospital, Ha Noi, Viet Nam
32. Anchalee Avihingsanon (PhD): HIV-NAT, Thai Red Cross AIDS Research Centre and Center of Excellence in Tuberculosis, Faculty of Medicine, Chulalongkorn University, Bangkok, Thailand
33. Andrew D Kerkhoff (MD): Division of HIV, Infectious Diseases and Global Medicine, Zuckerberg San Francisco General Hospital and Trauma Center, University of California San Francisco, San Francisco, California
34. Claudia M Denkinger (MD): FIND, Geneva, Switzerland & Division of Infectious Disease and Tropical Medicine, Heidelberg University Hospital and Faculty of Medicine, Heidelberg University, Heidelberg, Germany & German Centre for infection Research (DZIF), Partner site Heidelberg University Hospital, Heidelberg, Germany

35. Klaus Reither (PhD): Swiss Tropical and Public Health Institute, Allschwil, Switzerland & University of Basel, Basel, Switzerland
36. Lydia Nakiyingi (PhD): Infectious Diseases Institute, Makerere University, Kampala, Uganda
37. Prof Peter MacPherson (PhD): Public Health Group, Malawi-Liverpool-Wellcome Programme, Blantyre, Malawi & School of Health and Wellbeing, University of Glasgow, Glasgow, United Kingdom & Clinical Research Department, London School of Hygiene and Tropical Medicine, London, United Kingdom
38. Prof Graeme Meintjes (PhD): Department of Medicine, Faculty of Health Sciences, University of Cape Town, Cape Town, South Africa & Wellcome Center for Infectious Diseases Research in Africa, Institute of Infectious Disease and Molecular Medicine, University of Cape Town, Cape Town, South Africa
39. Morten Ruhwald (PhD): FIND, Geneva, Switzerland

Table of Contents

Table 1: Study tests that were routinely available in each study setting between 2019-2021 and whether they were communicated to the treating clinical teams 4
Table 2: Design of the Reference Standards 5
Table 3: STARD Checklist 6
Table 4: Reasons for being deemed unclassifiable against eMRS* 7
Table 5: Reasons for being deemed unclassifiable against CRS* 7
Table 6: TB Reference tests* conducted in the overall cohort 8
Table 7: Demographic and clinical characteristics, with stratification by inpatient versus outpatient site 9
Table 8: Clinical details of patients with "false positive" Urine-XPU results (against the CRS) 10
Table 9: Diagnostic accuracy of AlereLAM and Urine-XPU in predefined subgroups 11
Rifampicin probe results from Xpert Ultra tests on sputum and urine: 12
Supplementary Figure: Diagnostic yield per rapid test only in outpatients (A), those with CD4 counts of >200 cells per mL (B), and among those who were known to have survived 10 weeks since enrolment 13
Supplementary Methods 14
Supplementary Results 14

Table 1: Study tests that were routinely available in each study setting between 2019-2021 and whether they were communicated to the treating clinical teams

	Sputum Xpert Ultra	Sputum Culture	TB Blood Culture	Urine Xpert Ultra	AlereLAM
Malawi	• Available<br>• Results communicated to clinical team	• Available<br>• Results communicated to clinical team	• Available<br>• Results communicated to clinical team	• Restricted availability<br>• Results communicated to clinical team	• Available<br>• Results communicated to clinical team
South Africa	• Available<br>• Results communicated to clinical team	• Available<br>• Results communicated to clinical team	• Available<br>• Results communicated to clinical team	• Restricted availability<br>• Results communicated to clinical team	• Available<br>• Results communicated to clinical team
Uganda	• Available<br>• Results communicated to clinical team	• Available<br>• Results communicated to clinical team	• Available<br>• Results communicated to clinical team	• Not available<br>• Results not communicated to treating clinicians	• Available<br>• Results communicated to clinical team
Tanzania	• Available<br>• Results communicated to clinical team	• Available<br>• Results communicated to clinical team	• Available<br>• Results communicated to clinical team	• Available<br>• Results communicated to clinical team	• Available<br>• Results communicated to clinical team
Thailand	• Available<br>• Results communicated to clinical team	• Available<br>• Results communicated to clinical team	• Available<br>• Results communicated to clinical team	• Available<br>• Results communicated to clinical team	• Available<br>• Results communicated to clinical team
Vietnam	• Available<br>• Results communicated to clinical team	• Available<br>• Results communicated to clinical team	• Available<br>• Results communicated to clinical team	• Not available<br>• Results not communicated to treating clinicians	• Not available<br>• Results not communicated to treating clinicians
Zambia	• Available<br>• Results communicated to clinical team	• Available<br>• Results communicated to clinical team	• Available<br>• Results communicated to clinical team	• Restricted availability<br>• Results communicated to clinical team	• Available<br>• Results communicated to treating team

Table 2: Design of the Reference Standards

	eMRS	CRS
1-2 Sputum MGIT culture	X	X
1-2 Sputum LJ Culture	X	X
Blood culture	X	X
Sputum Xpert Ultra	X	X
Additional, clinically indicated non-study testing	X	X
2-3 months testing	X	X
Anti-TB therapy		X
Reference Standard Positive	Any of the eMRS tests is positive	Any of the eMRS tests is positive and/or TB treatment was started, and response to treatment with follow-up is documented
Reference Standard Negative	None of the eMRS tests is positive and at least one sputum culture is negative	None of the eMRS tests is positive and TB treatment was not started and the patient has no symptoms at 2-3 months
Unclassifiable	Neither reference standard positive nor reference standard negative	

CRS=Composite Reference Standard; eMRS=Extended Microbiological Reference Standard

Table 3: STARD Checklist

Section & Topic	No	Item	Reported on page #
**TITLE OR ABSTRACT**			
	**1**	Identification as a study of diagnostic accuracy using at least one measure of accuracy (such as sensitivity, specificity, predictive values, or AUC)	Done
**ABSTRACT**			
	**2**	Structured summary of study design, methods, results, and conclusions (for specific guidance, see STARD for Abstracts)	Done
**INTRODUCTION**			
	**3**	Scientific and clinical background, including the intended use and clinical role of the index test	6-7
	**4**	Study objectives and hypotheses	7
**METHODS**			
Study design	**5**	Whether data collection was planned before the index test and reference standard were performed (prospective study) or after (retrospective study)	10
Participants	**6**	Eligibility criteria	10
	**7**	On what basis potentially eligible participants were identified (such as symptoms, results from previous tests, inclusion in registry)	10
	**8**	Where and when potentially eligible participants were identified (setting, location and dates)	10
	**9**	Whether participants formed a consecutive, random or convenience series	10
Test methods	**10a**	Index test, in sufficient detail to allow replication	12
	**10b**	Reference standard, in sufficient detail to allow replication	12 & appendix pg 13
	**11**	Rationale for choosing the reference standard (if alternatives exist)	11
	**12a**	Definition of and rationale for test positivity cut-offs or result categories of the index test, distinguishing pre-specified from exploratory	12
	**12b**	Definition of and rationale for test positivity cut-offs or result categories of the reference standard, distinguishing pre-specified from exploratory	11
	**13a**	Whether clinical information and reference standard results were available to the performers/readers of the index test	12
	**13b**	Whether clinical information and index test results were available to the assessors of the reference standard	10 & 12
Analysis	**14**	Methods for estimating or comparing measures of diagnostic accuracy	12-13
	**15**	How indeterminate index test or reference standard results were handled	12
	**16**	How missing data on the index test and reference standard were handled	11
	**17**	Any analyses of variability in diagnostic accuracy, distinguishing pre-specified from exploratory	12-13
	**18**	Intended sample size and how it was determined	13
**RESULTS**			
Participants	**19**	Flow of participants, using a diagram	15
	**20**	Baseline demographic and clinical characteristics of participants	16-17 & appendix pg 10
	**21a**	Distribution of severity of disease in those with the target condition	16-17 & 21-23
	**21b**	Distribution of alternative diagnoses in those without the target condition	N/A
	**22**	Time interval and any clinical interventions between index test and reference standard	10-11
Test results	**23**	Cross tabulation of the index test results (or their distribution) by the results of the reference standard	12
	**24**	Estimates of diagnostic accuracy and their precision (such as 95% confidence intervals)	17-21 & appendix pg 12
	**25**	Any adverse events from performing the index test or the reference standard	N/A
**DISCUSSION**			
	**26**	Study limitations, including sources of potential bias, statistical uncertainty, and generalisability	26
	**27**	Implications for practice, including the intended use and clinical role of the index test	25-26
**OTHER INFO**	**28**	Registration number and name of registry	13
	**29**	Where the full study protocol can be accessed	13 & Appendix
	**30**	Sources of funding and other support; role of funders	14

Table 4: Reasons for being deemed unclassifiable against eMRS*

	Malawi	South Africa	Tanzania	Thailand	Uganda	Vietnam	Zambia	Total
No sputum culture was negative	14	50	3	4	1	0	3	75
Urine-XPU indeterminate/not available†	4	1	0	0	0	0	0	5
AlereLAM invalid‡	2	4	0	3	0	0	0	9

* Participants could meet multiple reasons for being deemed unclassifiable
† It was not possible to repeat Urine-XPU for an indeterminate result
‡ AlereLAM required 60ul to conduct the test and it was possible to repeat the test after an invalid result on the same specimen

eMRS=Extended Microbiological Reference Standard; Urine-XPU=Urine Xpert Ultra

Table 5: Reasons for being deemed unclassifiable against CRS*

	Malawi	South Africa	Tanzania	Thailand	Uganda	Vietnam	Zambia	Total
No sputum culture was negative	8	28	0	1	0	0	3	40
TB treatment started but died or no documented improvement	5	14	1	0	12	4	28	64
Not treated for TB and no resolution of symptoms documented at follow up	68	56	5	20	40	38	35	262
Urine-XPU indeterminate/not available†	3	1	0	0	1	0	2	9
AlereLAM invalid‡	2	4	0	3	0	0	0	9

* Participants could meet multiple reasons for being deemed unclassifiable
† It was not possible to repeat Urine-XPU for an indeterminate result
‡ AlereLAM required 60ul to conduct the test and it was possible to repeat the test after an invalid result on the same specimen

CRS=Composite Reference Standard; Urine-XPU=Urine Xpert Ultra

Table 6: TB Reference tests* conducted in the overall cohort

	Malawi (Inpatients & Outpatients)	South Africa (Inpatients only)	Tanzania (Outpatients only)	Thailand (Inpatients & Outpatients)	Uganda (Inpatients & Outpatients)	Vietnam (Inpatients & Outpatients)	Zambia (Inpatients & Outpatients)	Overall
All tests: median (range; IQR)	4 (0-7; 4-7)	4 (1-14; 2-6)	4 (3-7; 4-4)	4 (1-7; 4-5)	4 (3-7, 4-4)	7 (2-7, 4-7)	4 (0-8, 4-4)	4 (0-14, 4-6)
In eMRS-positive, total tests: median (range)	4 (2-7)	4 (1-14)	4 (4-7)	4 (4-7)	4 (4-4)	4 (4-7)	4 (3-7)	4 (1-14)
In eMRS-negative, total tests: median (range)	4 (1-7)	5 (2-13)	4 (3-7)	4 (3-7)	4 (3-7)	7 (2-7)	4 (3-8)	4 (1-13)
MTB-prevalence: n/d (%)	28/349 (8.0)	54/199 (27.1)	49/242 (20.2)	7/131 (5.3)	41/247 (16.6)	33/177 (18.6)	51/360 (14.2)	263/1705 (15.4)
Without micro confirmed MTB, but treated for TB n/d (%)	27/349 (7.7)	24/199 (12.1)	18/242 (7.4)	9/131 (6.9)	69/247 (27.9)	5/177 (2.8)	107/360 (29.7)	259/1705 (15.2)

* TB Reference test refers to mycobacterial culture (e.g. solid culture, liquid culture on any specimen) or Xpert Ultra (on any specimen excluding urine). Smear microscopy was also done on certain specimens, but not counted towards the reference standard as per protocol. Reference tests were captured between enrolment and the 2-3 month follow up visit, as per the protocol.

Table 7: Demographic and clinical characteristics, with stratification by inpatient versus outpatient site

	All (n=1602)*	Inpatient (n=665)	Outpatient (n=937)	p value
Age, median (IQR)	40 (33-48)	39 (32-47)	41 (34-49)	
Female, no (%)	838 (52.3)	345 (51.9)	493 (52.6)	
ART status				
• Treatment interruption, no (%)	88 (5.5)	72 (10.8)	16 (1.7)	<0.0001
• Currently on ART, no (%)	1246 (77.8)	452 (68.0)	794 (84.7)	<0.0001
• ART-naïve, no (%)	250 (15.6)	128 (19.2)	122 (13.0)	0.0007
• ART status unknown, no (%)	18 (1.1)	13 (2.0)	5 (0.5)	
CD4 count				
• Median (IQR)	374 (138-630)	188 (55-421)	500 (269-702)	<0.0001
• ≤200cells/µl, no (%)	504 (31.5)	334 (50.2)	170 (18.1)	<0.0001
• >200cells/µl, no (%)	1082 (67.5)	324 (48.7)	758 (80.9)	<0.0001
• Unknown	16 (1.0)	7 (1.1)	9 (1.0)	
History of TB prior, no (%)	421 (26.3)	168 (25.3)	253 (27.0)	0.436
Seriously ill criteria†	698 (43.6)	386 (58.0)	312 (33.3)	<0.0001
WHO Symptom Screen‡	1491 (93.0)	560 (84.2)	931 (99.4)	<0.0001
Vital status at 10 weeks				
• Alive	1379 (86.1)	484 (72.8)	895 (95.5)	
• Died	115 (7.2)	105 (15.8)	10 (1.1)	<0.0001
• LTFU	108 (6.3)	76 (11.4)	32 (3.4)	
eMRS status				
• Positive	254 (15.9)	130 (19.5)	124 (13.2)	0.0007
• Negative	1348 (84.1)	535 (80.5)	813 (86.8)	

* Table includes those participants that were classifiable by eMRS
† Criteria based on WHO danger signs: BMI<18.5kg/m2 or RR>30 or systolic BP <90 or HR>120 or inability to walk unaided
‡ Any of the four symptoms present: cough, loss of weight, night sweats or fever

ART=Antiretroviral Therapy; BMI=Body Mass Index; BP=Blood Pressure; eMRS=Extended Microbiological Reference Standard; HR=Heart Rate; IQR=Interquartile Range; RR=Respiratory Rate; TB=Tuberculosis; WHO=World Health Organisation

Table 8: Clinical details of patients with "false positive" Urine-XPU results (against the CRS)

	Urine-XP quantitative result	Demographics (age, sex)	CD4	eMRS	Setting	TB symptoms	Previous TB	ART status	CXR	Seriously ill criteria	AlereLAM results	Sputum results	Other microbiological results	Outcome
TB036070019	Low	43, Female	1183	Neg	Outpatient	Yes	No	Currently on ART	Normal	BMI≤18.5	Both negative	Neg	None captured	Improved without TB treatment
TB036070020	Trace	64, Female	652	Neg	Outpatient	Yes	No	Currently on ART	"Pneumonia" Right sided infiltrate	None	Day 1 positive	Neg	None captured	Improved without TB treatment
TB036070130	Trace	34, Female	681	Neg	Outpatient	Yes	Yes	Currently on ART	"TB likely" Left sided cavitation	None	Both negative	Neg	None captured	Improved without TB treatment
TB036070142	Trace	40, Male	652	Neg	Outpatient	Yes	Yes	Currently on ART	"Pneumonia" Right sided infiltrate	None	Day 2 positive	Neg	None captured	Improved without TB treatment
TB036070145	Trace	43, Female	730	Neg	Outpatient	Yes	No	Currently on ART	"TB likely" Right sided infiltrate and cavitation	None	Both negative	Smear 1+	None captured	Improved without TB treatment
TB036070147	Trace	52, Male	341	Neg	Outpatient	Yes	No	Currently on ART	"Pneumonia or atypical TB" Right sided lymphadenopathy	None	Both negative	Neg	None captured	Improved without TB treatment
TB036070156	Trace	50, Female	811	Neg	Outpatient	Yes	Yes	Currently on ART	"TB likely" Left sided infiltrate and cavitation	None	Both negative	Neg	None captured	Improved without TB treatment
TB036110020	Trace	49, Male	525	Neg	Outpatient	Yes	No	Currently on ART	Normal	None	Both negative	Neg	None captured	Improved without TB treatment

CRS=Composite Reference Standard; CXR=Chest Xray; eMRS=Extended Microbiological Reference Standard; TB=Tuberculosis

Table 9: Diagnostic accuracy of AlereLAM and Urine-XPU in predefined subgroups

Index	Group	Test	N	TP	FN	Sens_denom	FP	TN	Spec_denom	Sens	Sens_LL	Sens_UL	Spec	Spec_LL	Spec_UL	PPV	NPV
1	eMRS, all	Alere	1602	78	176	254	130	1218	1348	30,71	25,36	36,63	90,36	88,67	91,82	37,50	87,37
2	eMRS, all	Urine-XPU	1602	83	171	254	27	1321	1348	32,68	27,20	38,67	98,00	97,10	98,62	75,45	88,54
3	eMRS, CD4≤200	Alere	504	64	64	128	53	323	376	50,00	41,47	58,53	85,90	82,02	89,06	54,70	83,46
4	eMRS, CD4≤200	Urine-XPU	504	67	61	128	5	371	376	52,34	43,75	60,80	98,67	96,93	99,43	93,06	85,88
5	eMRS, CD4>200	Alere	1082	13	108	121	76	885	961	10,74	6,39	17,52	92,09	90,21	93,64	14,61	89,12
6	eMRS, CD4>200	Urine-XPU	1082	14	107	121	22	939	961	11,57	7,02	18,49	97,71	96,56	98,48	38,89	89,77
7	eMRS, Inpatients only	Alere	665	54	76	130	69	466	535	41,54	33,43	50,13	87,10	84,00	89,68	43,90	85,98
8	eMRS, Inpatients only	Urine-XPU	665	59	71	130	6	529	535	45,38	37,08	53,95	98,88	97,58	99,49	90,77	88,17
9	eMRS, Outpatients only	Alere	937	24	100	124	61	752	813	19,35	13,37	27,19	92,50	90,48	94,11	28,24	88,26
10	eMRS, Outpatients only	Urine-XPU	937	24	100	124	21	792	813	19,35	13,37	27,19	97,42	96,08	98,30	53,33	88,79
11	CRS, all	Alere	1359	136	310	446	45	868	913	30,49	26,40	34,92	95,07	93,47	96,30	75,14	73,68
12	CRS, all	Urine-XPU	1359	94	352	446	8	905	913	21,08	17,55	25,10	99,12	98,28	99,56	92,16	72,00
13	CRS, CD4≤200	Alere	399	90	113	203	14	182	196	44,33	37,67	51,21	92,86	88,37	95,70	86,54	61,69
14	CRS, CD4≤200	Urine-XPU	399	70	133	203	0	196	196	34,48	28,11	41,13	100,00	98,08	100,00	100,00	59,57
15	CRS, CD4>200	Alere	951	45	192	237	31	683	714	18,99	14,50	24,46	95,66	93,90	96,92	59,21	78,06
16	CRS, CD4>200	Urine-XPU	951	22	215	237	8	706	714	9,28	6,21	13,65	98,88	97,80	99,43	73,33	76,66
17	CRS, Inpatients only	Alere	499	86	131	217	13	269	282	39,63	33,36	46,27	95,39	92,27	97,29	86,87	67,25
18	CRS, Inpatients only	Urine-XPU	499	60	157	217	0	282	282	27,65	22,13	33,95	100,00	98,66	100,00	100,00	64,24
19	CRS, Outpatients only	Alere	860	50	179	229	32	599	631	21,83	16,97	27,63	94,93	92,93	96,39	60,98	76,99
20	CRS, Outpatients only	Urine-XPU	860	34	195	229	8	623	631	14,85	10,82	20,03	98,73	97,52	99,36	80,95	76,16

eMRS=Extended Microbiological Reference Standard; FN=False Negative; FP=False Positive; LL=Lower Limit of the 95% Confidence Interval; NPV=Negative Predictive Value; PPV=Positive Predictive Value; Sens=Sensitivity; Spec=Specificity; TN=True Negative; TP=True Positive; UL=Upper Limit of the 95% Confidence Interval; Urine-XP=Urine Xpert Ultra

A

	Urine Rif resistance detected	Urine Rif sensitive	Urine Trace/Indeterminate	No MTB detected on urine
Sputum Rif resistance detected	4	0	0	6
Sputum Rif sensitive	0	42	10	64
Sputum Trace/Indeterminate	0	2	5	50
No MTB detected on sputum	2	33	20	N/A

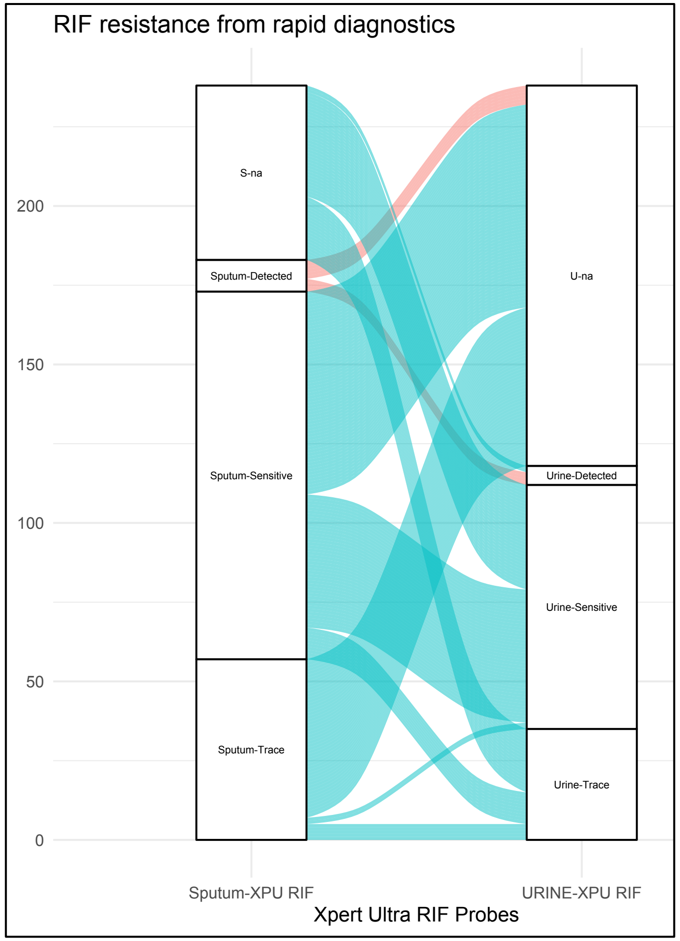

B

Rifampicin probe results from Xpert Ultra tests on sputum and urine:

A) Table demonstrating distribution of Rif-probe results between study urine and sputum Xpert Ultra ("rapid tests"), in those that were rapid test positive

B) Alluvium plot in those with either Sputum-XPU or Urine-XPU-confirmed MTB demonstrating the agreement between Rif-probe results, where pink demonstrates those with Rifampicin-resistance detected and blue in those without Rifampicin-resistance detected.
S-na = no MTB was detected on the sputum result and U-na = no MTB was detected on the urine result.

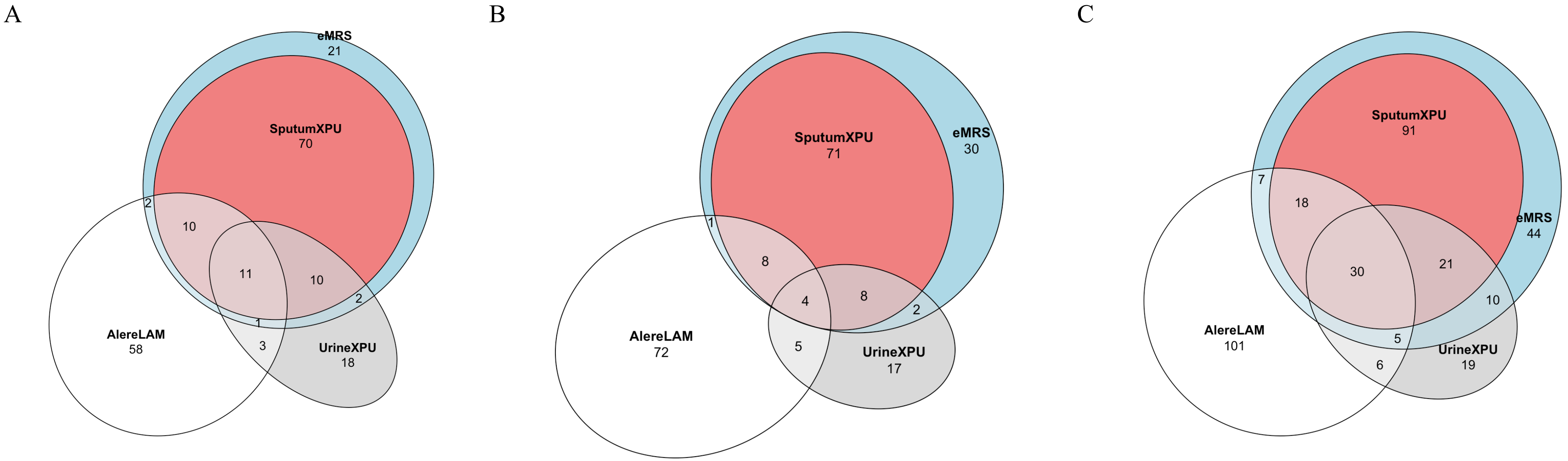

A

Diagnostic yield
Urine Xpert Ultra = 24 / 128 (18.8%)
AlereLAM = 24 / 128 (18.8%)
Sputum Xpert Ultra = 101 / 128 (78.9%)
All 3 tests = 107 / 128 (83.6%)

B

Diagnostic yield
Urine Xpert Ultra = 14 / 124 (11.3%)
AlereLAM = 13 / 124 (10.5%)
Sputum Xpert Ultra = 91 / 124 (73.4%)
All 3 tests = 94 / 124 (75.8%)

C

Diagnostic yield
Urine Xpert Ultra = 66 / 225 (29.3%)
AlereLAM = 60 / 225 (26.7%)
Sputum Xpert Ultra = 160 / 225 (71.1%)
All 3 tests = 181 / 225 (80.4%)

Supplementary Figure: Diagnostic yield per rapid test only in outpatients (A), those with CD4 counts of >200 cells per µL (B), and among those who were known to have survived 10 weeks since enrolment

Supplementary Methods

Reference standard testing

Cultures were with both mycobacteria growth indicator tube (MGIT; Becton Dickinson, Franklin Lakes, NJ, USA) liquid culture, and with solid culture on Löwenstein-Jensen medium. Identification of MTB in solid and liquid cultures was confirmed with antigen detection (MPT64 antigen detection and/or MTBDRplus, MTBC and CM/AS line probe assays, Hain Lifescience, Nehren, Germany). Blood cultures were done in BACTEC^{TM} Myco/F Lytic culture vials (Becton Dickinson, Franklin Lakes, NJ, USA). Incubation periods were for a maximum of 6-8 weeks.

Supplementary Results

Demographics: inpatients versus outpatients

A total of 41.5% (n=665/1602) of participants classifiable in the eMRS analysis were recruited from inpatient settings. Inpatients were more likely to not be taking ART at enrollment, had lower CD4 counts (median 188cells/µl in inpatients; 500cells/µl in outpatients) and more met the "seriously ill" criteria (58.0% in inpatients; 33.3% in outpatients). More inpatients were eMRS-positive (19.5%; compared to 13.2% in outpatients), and this group was more likely to die within ten weeks (15.8%; compared to 1.1% in outpatients).

Overall diagnostic yield

In the AlereLAM-positive, eMRS-negative group, 22/131 (16.8%) had CD4 ≤50cells/ul, 75/131 (57.3%) were treated for TB with 60/75 (80.0%) surviving to ten weeks and 12/75 dying (16.0%). Of the 56/131 who were not treated for TB, 47/56 (83.9%) survived to ten weeks and 4/56 (7.1%) died. In the Urine-XPU-positive, eMRS-negative group, all had CD4 greater than 50 cells/ul, 15/27 (55.6%) were treated for TB, with all surviving to ten weeks. Of the 12/27 who were not treated for TB, 10/12 were known to have survived to ten weeks, none were known to have died, but two were lost to follow up.

TB outcomes and prognostic value of AlereLAM

In univariable analysis, participants with positive AlereLAM results (16.8%, 95% CI: 12.3-22.6%), were significantly more likely to have died by 10-weeks compared to those with negative AlereLAM results (7.1%, 95%CI: 5.9-8.7%), (average effect: +9.7%, 95% CI: +4.4% to +15.0%; odds ratio: 2.6, 95% CI: 1.7-4.00). In the random effects multivariable model, for participants with microbiologically-confirmed TB taking ART, with other variables held at their mean (CD4, age), participants with positive AlereLAM remained at higher risk of death (adjusted odds ratio: 1.8, 95% CI: 1.1-3.1), with a conditional average effect on death of +4.4% percentage points (95% CI: +0.1% to 13.0%).

CLINICAL TRIAL PROTOCOL

Protocol Title

Prospective Multicentre Evaluation of the Accuracy and Diagnostic Yield of the Fujifilm SILVAMP TB LAM Test for the Diagnosis of Tuberculosis in People Living with HIV

Short title

FujiLAM prospective evaluation

Protocol Version Number:

v.5.0

Date:

16JULY2020

Disease Programme:

TB

Regulatory Agency Identifying Number(s):

N/A

Clinicaltrials.gov Registration Number:

NCT04089423

Because diagnosis matters

FIND
Campus Biotech
Chemin des Mines 9
1202 Geneva, Switzerland
T: +41 (0)22 710 05 90

Confidentiality Statement:

The information contained in this document, especially unpublished data, is the property of FIND (or under its control) and may not be reproduced, published or disclosed to others without prior written authorization from FIND.

CONFIDENTIAL

FIND

Table of Contents

Institutions/Organizations/Partners Involved in the Trial* 5

Signature Page (Sponsor) 7

Statement of Principal Investigator 8

Protocol History/Amendment Summary 9

List of Abbreviations and Acronyms 10

Protocol Synopsis 12

Schedule of Activities 17

Specimen Flow 18

1 Introduction 19

1.1 Trial Rationale 19

1.2 Background 20

1.3 Benefit/Risk Assessment 21

2 Trial Objectives and Endpoints 22

3 Trial Design 23

3.1 General Design 23

3.2 Scientific Rationale for Trial Design 23

3.3 End of Trial Definition 24

3.4 Trial Population and Eligibility 24

3.5 Inclusion Criteria 24

3.6 Exclusion Criteria 25

3.7 Early exclusions 25

4 Trial Intervention 25

4.1 Investigational Product 25

4.1.1 Investigational Product: Fujifilm SILVAMP TB LAM (FujiLAM) test 25

4.1.2 Comparator: Alere Determine™ TB LAM (AlereLAM) 26

4.1.3 Investigational product: VISITECT® CD4 Advanced Disease Rapid Test 26

4.2 Preparation/Handling/Storage/Accountability 27

4.3 Minimisation of Error and Bias 28

5 Participant Discontinuation/Withdrawal 29

5.1 Participant Discontinuation/Withdrawal from the Trial 29

5.2 Lost to Follow Up 30

6 Trial Procedures 31

6.1 Specimen Collection, Handling, Storage 31

6.2 Reference Standard Test and Investigational Test Procedures 36

CONFIDENTIAL

6.3 Comparator test procedure ... 38
6.4 Safety Assessments ... 38
7 Safety and Incident Reporting ... 38
7.1 Adverse Events and Serious Adverse Events ... 39
7.1.1 Time Period for Collecting SAE Information ... 39
7.1.2 Reporting and Follow up of SAEs ... 39
7.1.3 Medical Device Incidents (including Malfunctions) ... 39
7.1.4 Time Period for Detecting Medical Device Incidents ... 40
7.1.5 Follow-up of Medical Device Incidents ... 40
7.1.6 Reporting of Medical Device Incidents to FIND ... 40
7.1.7 Regulatory Reporting Requirements for Medical Device Incidents ... 40
8 Statistical Considerations ... 41
8.1 Populations for Analyses ... 41
8.2 Statistical Analyses ... 41
8.3 Sample Size Determination ... 44
8.4 Other analyses ... 45
8.5 Planned Interim Analyses ... 45
8.6 Statistical software ... 45
9 Regulatory and Ethical Considerations ... 45
9.1 Regulatory and Ethics Approvals ... 45
9.2 Financial Disclosure ... 46
9.3 Informed Consent Process ... 46
9.4 Data Protection ... 47
10 Data Handling and Record Keeping ... 47
10.1 Source Data and Source Documents ... 48
10.2 Data Management ... 48
11 Quality Management ... 49
11.1 Quality Control (monitoring) ... 49
11.2 Quality Assurance (auditing) ... 49
11.3 Trial and Site Closure ... 50
12 Publication Policy ... 50
13 References ... 50
14 Appendices ... 51
Appendix 1: Safety Definitions and Reporting ... 51
Appendix 2: Incident Definition and Reporting ... 53

CONFIDENTIAL

Institutions/Organizations/Partners Involved in the Trial*

FIND (Sponsor)

Campus Biotech
Chemin des Mines 9
1202 Geneva
Switzerland

Centre for Infectious Disease Research in Zambia (Investigational Site)

Plot 34620, Off Alick Nkhata Road, Mass Media, Lusaka
P.O. Box 34681
Lusaka, 10101
Zambia

Infectious Diseases Institute (Investigational Site)

College of Health Sciences, Makerere University,
P.O. Box 22418, Kampala
Uganda

Malawi-Liverpool-Wellcome Trust Clinical Research Programme (Investigational Site)

College of Medicine
Queen Elizabeth Central Hospital,
P.O. Box 30096, Chichiri, Blantyre 3
Malawi

Swiss TPH (1) & Ifakara Health Institute (2) (Investigational Site)

1. Swiss Tropical and Public Health Institute
Socinstrasse 57, 4051 Basel, Switzerland
2. Ifakara Health Institute
Kiko Avenue, Mikocheni, P.O. Box 78 373, Dar es Salaam
Tanzania

University of Cape Town (Investigational Site)

CIDRI-Africa
Institute of Infectious Disease and Molecular Medicine
Faculty of Health Sciences
University of Cape Town
Anzio Road, Observatory, Cape Town, 7925
South Africa

Viet Tiep Hospital (Investigational Site)

No1 Nha Thuong Street,
Le Chan District, Hai Phong City
Vietnam

Fujifilm Corporation (Manufacturer)

577 Ushijima, Kaisei-Machi, Ashigarakami-gu
Kanagawa,
258-8577
Japan

Omega Diagnostics (Manufacturer)
Omega House,
Hillfoots Business Village,
Alva,
FK12 5DQ Scotland,
United Kingdom

Zeptometrix (Biobank repository)

872 Main St
Buffalo, NY 14202
USA

*Terms of references and nature of agreements are available from FIND on request.

CONFIDENTIAL

CONFIDENTIAL

Signature Page (Sponsor)

We, the undersigned, have developed, reviewed and approved this protocol, including appendices. We will supervise and coordinate the clinical trial according to the principles outlined in the Declaration of Helsinki and Good Clinical Practice and in compliance with applicable regulatory requirements.

HEAD OF TB PROGRAMME

Name: Morten Ruhwald

Signature: ______________________________ Date: ____________________

DD/MMM/YYYY

TRIAL MANAGER

Name: Rita Szekely

Signature: ______________________________ Date: ____________________

DD/MMM/YYYY

SENIOR MEDICAL OFFICER

Name: Pamela Nabeta

Signature: ______________________________ Date: ____________________

DD/MMM/YYYY

HEAD OF DATA SERVICES & BIOBANKING

Name: Stefano Ongarello

Signature: ______________________________ Date: ____________________

DD/MMM/YYYY

CONFIDENTIAL

CONFIDENTIAL

Statement of Principal Investigator

In signing this page, I, the undersigned, agree to conduct the trial according to the protocol and ICH-GCP E6 (R2) guidelines and in compliance with applicable regulations.

I will ensure that the requirements relating to obtaining Institutional Review Board (IRB)/ Independent Ethics Committee (IEC) review and approval are met. I will promptly report to the IRB/IEC any and all changes in the research activities covered by this protocol.

I have sufficient time to properly conduct and complete the trial within the agreed trial period and I have adequate resources (staff and facilities) for the foreseen duration of the trial.

I am responsible for supervising any individual or party to whom I delegate trial related duties and functions conducted at the trial site. Further, I will ensure this individual or party is qualified to perform those trial-related duties and functions.

I certify that key individuals involved with the conduct of this trial, including myself, have completed GCP training and, if applicable, Human Subjects Protection Training.

I understand that all information obtained during the conduct of the trial with regard to the subjects' state of health will be regarded as confidential. No participant's names or personal identifying information may be disclosed. All participant data will be anonymized and identified by assigned numbers on all Case Report Forms, laboratory samples and other trial related information (such as essential documents) forwarded to FIND. Monitoring and auditing by FIND, and inspection by the appropriate regulatory authority(ies), will be permitted.

I will maintain confidentiality of this protocol and all other related investigational materials. Information taken from the trial protocol may not be disseminated or discussed with a third party without the express consent of FIND.

Name of Principal Investigator: ___________________________________
(Print)

Name:

Signature: _____________________________________ Date: ________________________

DD/MMM/YYYY

CONFIDENTIAL

Protocol History/Amendment Summary

Version number	Release date	Comments
v.1.0	24MAY2019	Initial version
v2.0	17JUL2019	Modification in the protocol regarding the requirement of two separate signatures on the consent form (Section 9.3)
v. 3.0	13SEP2019	Addition of the Clinicaltrials.gov registration number<br>Deletion of the DRAFT watermark on the first page
v. 4.0	12DEC2019	Addition of storage of X-ray pictures for database and artificial intelligence project<br>Change of Xpert/Ultra to Ultra on some of the Figures<br>Change of VISITECT® CD4 to VISITECT® CD4 Advanced Disease<br>Addition of the definition of incident
v5.0	16JUL2020	Changes in the sample size calculation with two TB prevalence options<br>Change in the number of Asian sites and addition of a potential site from Europe

CONFIDENTIAL

FIND

List of Abbreviations and Acronyms

Abbreviation/acronym	Meaning
AE	Adverse Event
ART	Antiretroviral Therapy
ATT	Antitubercular Therapy
AlereLAM	Alere Determine™ TB LAM Ag
CAD4TB	Computer-Aided Detection for Tuberculosis
CI	Confidence Interval
CD4	Cluster of differentiation 4
CRF	Case Report Form
CRS	Composite reference standard
DM	Data Management
eMRS	Extended microbiological reference standard
EPTB	Extrapulmonary TB
FU	Follow-up
FujiLAM	Fujifilm SILVAMP TB LAM
GCP	Good Clinical Practice
GCLP	Good Clinical Laboratory Practice
GDoP	Good Documentation Practice
HIV	Human immunodeficiency virus
ICF	Informed Consent Form
IDMC	Independent Data-Monitoring Committee
IEC	Independent Ethics Committee
ICH	International Council on Harmonisation
IRB	Institutional Review Board
ISF	Investigator Site File
ISO	International Organisation for Standardization
ITT	Intention-to-test population

CONFIDENTIAL

CONFIDENTIAL

LAM	Lipoarabinomannan
MTB	*Mycobacterium tuberculosis*
MRS	Microbiological reference standard
PCP	Partially Compliant Population
pCRF	Paper Case Report Form
PLHIV	People living with HIV
POC	Point-of-care
PP	Protocol Population
PTB	Pulmonary TB
QA	Quality Assurance
QC	Quality Control
QMS	Quality Management System
RA	Regulatory Authority
RBM	Risk Based Monitoring
RM	Risk Management
SAP	Statistical Analysis Plan
SAE	Serious Adverse Event
SOA	Schedule of Activities
SOP	Standard Operating Procedure
TB	Tuberculosis
TMF	Trial Master File
WHO	World Health Organisation
Ultra	Xpert Ultra MTB/RIF (Cepheid)

CONFIDENTIAL

CONFIDENTIAL

**Title**	Prospective Multicentre Evaluation of the Accuracy and Diagnostic Yield of the Fujifilm SILVAMP TB LAM Test for the Diagnosis of Tuberculosis in People Living with HIV
**Short title**	FujiLAM prospective evaluation
**Protocol version and date**	V. 4.0
**Background and rationale**	Traditional diagnostic methods, such as sputum culture or sputum smear microscopy, are slow or low in sensitivity and yield. Newer techniques for testing of sputum specimens, such as sequencing or Xpert® MTB/RIF, require specialized facilities, are costly, or are otherwise inaccessible to populations at greatest risk of contracting TB. Moreover, TB is harder to diagnose in PLHIV, since many of the patients have extrapulmonary TB (EPTB, ~25%) or often paucibacillary TB. TB in PLHIV is almost certain to be fatal if undiagnosed or left untreated.<br><br>The marketed Alere Determine TB LAM Ag (AlereLAM) is a simple urine-based test and can be used in severely ill PLHIV but has low sensitivity, limiting its overall utility. A rapid, easy to use, highly-sensitive test is urgently needed. To address this unmet diagnostic need, Fujifilm and FIND have developed the biomarker-based Fujifilm SILVAMP TB LAM (FujiLAM) test.<br><br>The FujiLAM test is based on the sensitive and specific detection of Lipoarabinomannan (LAM) in the urine of patients to diagnose active TB. The superior analytical sensitivity of FujiLAM compared to AlereLAM relies on the use of two novel monoclonal antibodies with particular binding specificity to MTB LAM-epitopes in the urine of TB patients. The expected cut-off of detection of the Fuji-TB test is ~30 pg/ml which is ~30-times below that of the marketed AlereLAM.<br><br>In resource poor settings there is limited access to CD4+ T cells measurement to aid management of patients with pre-diagnosed HIV infection. Flow cytometry is the established reference method for CD4 testing and is often only available at centralised laboratories. Accurate, inexpensive POC tests are needed that can provide CD4+ T cell estimates at the primary healthcare level. VISITECT® CD4 Advanced Disease is a rapid, affordable and instrument-free test that provides actionable results at point of care. Such a test will be of critical importance, if other tests for co-morbidities only have a recommendation for subsets of the PLHIV, i.e. AlereLAM is only recommended by the WHO currently for patients with CD4 <100 and an updated recommendation will likely expand the recommendation to patients with CD4 <200.<br><br>The goal of this study is to confirm the performance of both FujiLAM and VISITECT® CD4 Advanced Disease on prospectively collected, fresh specimens.<br><br>The data gathered from this trial, will form part of the dossier to be submitted to WHO for review.

**Reference standards**	**Microbiological reference standard (MRS):**<br>Patients will be considered positive using MRS if they have positive results by one or more of the following tests: MGIT culture, LJ culture and Ultra on sputum; Ultra on urine; Mycobacterial blood culture. Patients will be considered negative on MRS if they have negative results on all listed tests for which results are available.<br>**Extended microbiological reference standard (eMRS):**<br>Patients will be considered positive on eMRS if they are positive on MRS or if they have positive results on any additional mycobacterial culture or Ultra from other respiratory and/or non-respiratory samples (e.g. pleural fluid, tissue biopsy, etc.) that were performed based on routine clinical indication. Patients will be considered negative on eMRS if they have negative results on all listed tests for which results are available.<br>**Composite reference standard (CRS):**<br>Patients will be considered positive on CRS if they are positive based on eMRS or if started on anti-TB treatment based on clinical grounds (clinical/radiographic features) or both with response to TB treatment. Patients will be considered negative on CRS if they have negative results on all listed tests for which results are available and are not started on anti-TB treatment.
**Primary objective(s)**	1.1 To determine the diagnostic accuracy of FujiLAM for TB detection among PLHIV using an eMRS<br>1.2 To determine the diagnostic accuracy of FujiLAM for TB detection among PLHIV using a CRS<br>1.3 To determine the diagnostic yield of FujiLAM, AlereLAM, Smear and Ultra (sputum, urine) among all patients identified as having TB by eMRS (on Day 1 specimens).

CONFIDENTIAL

**Secondary objective(s)**	2.1. To determine the diagnostic accuracy of FujiLAM for TB detection among PLHIV using a MRS.<br>2.2. To determine the diagnostic yield of FujiLAM, AlereLAM, Smear and Ultra (sputum, urine) among all patients identified by MRS (on Day 1 specimens).<br>2.3. To determine the diagnostic accuracy of FujiLAM across predefined subgroups using a MRS, eMRS and CRS separately.<br>2.4. To determine the diagnostic accuracy of FujiLAM of Day 2 early morning urine using a MRS, eMRS and CRS separately.<br>2.5. To determine the diagnostic accuracy of FujiLAM of Day 2 early morning urine across subgroups using a MRS, eMRS and CRS separately.<br>2.6. To determine the diagnostic accuracy of AlereLAM in the same way as described in objectives 1.1, 1.2 and 2.1, 2.3 and estimate the difference to that of FujiLAM.<br>2.7. To determine the yield of FujiLAM over (incremental yield) or in combination with (combined yield) Smear and Ultra (sputum) among *eMRS-positive patients*.<br>2.8. To determine the association of positive FujiLAM results with mortality (only at sites where feasible)<br>2.9. To determine the diagnostic accuracy of VISITECT® CD4 Advanced Disease test for detection of a CD4 count ≤200 cells/µl, using WHO pre-qualified cytometers as the reference standard.<br>2.10. To assess the feasibility, ease of use of the FujiLAM test at POC
**Primary endpoints (outcomes)**	1.1. Point estimates of sensitivity and specificity of FujiLAM, with 95% confidence intervals, using the defined eMRS.<br>1.2. Point estimates of sensitivity and specificity of FujiLAM, with 95% confidence intervals, using the defined CRS.<br>1.3. Diagnostic yield, with 95% confidence interval, of FujiLAM test among eMRS positive patients and AlereLAM, Smear and Ultra (sputum, urine) as comparators (on Day 1 specimens).

**Secondary endpoints (outcomes)**	2.1. Point estimates of sensitivity and specificity of FujiLAM, with 95% confidence intervals, using the defined MRS.<br>2.2. Diagnostic yield, with 95% confidence interval, of FujiLAM test among MRS positive patients and AlereLAM, Smear and Ultra (sputum, urine) as comparators (on Day 1 specimens).<br>2.3. Point estimates of sensitivity and specificity of FujiLAM, with 95% confidence intervals, across predefined subgroups, using the defined MRS, eMRS and CRS separately.<br>2.4. Point estimates of sensitivity and specificity, with 95% confidence intervals, of FujiLAM of Day 2 early morning urine, using the defined MRS, an eMRS and a CRS separately.<br>2.5. Point estimates of sensitivity and specificity, with 95% confidence intervals, of FujiLAM of Day 2 early morning urine across subgroups, using the defined MRS, eMRS and CRS separately.<br>2.6. Point estimates of sensitivity and specificity, with 95% confidence intervals, of AlereLAM in the same way as described in endpoints 1.1, 1.2, and 2.1, 2.3.<br>2.7. Yield of FujiLAM over (incremental yield) or in combination with (combined yield) Smear and Ultra (sputum) among eMRS-positive patients.<br>2.8. Kaplan-Meier survival analysis for the outcome of death within 2-3 months by TB and FujiLAM status (only at sites where feasible)<br>2.9. Point estimates of sensitivity and specificity, with 95% confidence intervals, of VISITECT® CD4 Advanced Disease test, using cytometry as the reference standard.<br>2.10. Time to proficiency, implementation issues, design related issues at POC
**Trial design**	This is a prospective, multicentre cohort study in which the accuracy and the diagnostic yield of the FujiLAM test will be assessed using a microbiological reference standard, an extended microbiological reference standard and a composite reference standard among inpatient and outpatient PLHIV.
**Trial sites/setting**	5 sites in sub-Saharan Africa<br>1 or 2 sites in Asia<br>Optional: 1 site in Europe<br>Outpatient and inpatient settings
**Trial population**	Adult PLHIV
**Sample Size**	Enrolment will target to reach 233 Confirmed TB cases. The total sample size being enrolled will depend on the average prevalence observed during enrolment.<br>If TB prevalence is 20% the total sample size is of 1440 PLHIVs across sites (with 20% LTFU). If TB prevalence is 15% the total sample size is of 1980 PLHIV across sites (with 20% LTFU). The enrolment targets an approx. 50%-50% distribution of outpatients and inpatients across all sites.

Eligibility criteria	Inclusion criteria:<br>• Adult PLHIV (≥18 years), irrespective of their CD4 count and antiretroviral therapy (ART) status, at risk of having pulmonary and/or extra-pulmonary TB<br>• Inpatients: irrespective of TB symptoms<br>• Outpatients: at least one of the symptoms suggestive of TB (as defined by WHO*)<br>• written informed consent<br>• willingness to have a trial follow-up visit at 2-3 and 6 months after enrolment (e.g. not planning to relocate)<br><br>Exclusion criteria:<br>• Current anti-TB treatment *<br>• Any anti-TB treatment within 60 days prior to enrolment<br>• Any isoniazid preventive therapy within 6 months prior to enrolment<br><br>* Patients starting anti-TB treatment at the time of enrolment will not be excluded from the trial provided that all trial specimens are collected before starting the 3rd dose of treatment.<br><br>Follow-up:<br>• At 2-3 months for participants with a negative eMRS<br>• At 6 months for participants with a positive index test in the context of a negative CRS
**Trial duration**	18 months
**Time schedule**	Trial setup (protocol, contracts, IRB, etc.): months 1-4<br>Enrolment: months 5-9<br>MRS results and 2-3-months FU: months 7-11<br>Results on 2-3-months FU samples: months 9-13<br>6-months FU and discrepant resolution: months 11-15<br>Results on 6-months FU samples: months 13-17<br>Analysis and report/manuscript preparation: months 13-18

* Current cough, fever, weight loss, night sweats

Schedule of Activities

Procedure	Enrolment		Follow-up		Notes
	Day 1	Day 2	2-3 month after enrolment[1]	6 months after enrolment[2]	
Inclusion and exclusion criteria	X				TB symptoms only apply to outpatient settings
Informed consent	X				
Clinical questionnaire	X		X	X	
Chest X-ray	X				
Blood collection & testing (blood culture, CD4 flow cytometry)	X				
Fingerstick collection (VISITECT® CD4 Advanced Disease)	X				
Urine collection & testing (FujiLAM, AlereLAM, Ultra on conc. urine)	X[3]	X		X	Only FujiLAM and AlereLAM tests will be performed from a Day 2 early morning urine
Sputum collection & testing (smear, LJ, MGIT, Ultra)	X	X	X[4]	X[4]	For patients who are unable to provide expectorated sputum, an attempt will be made to obtain induced sputum
Record results of tests performed as per routine which contribute to clinical decision of current episode	X	X	X	X	Any additional mycobacterial culture or Xpert (MTB/RIF or Ultra) from other respiratory or non-respiratory samples (e.g. pleural fluid, tissue biopsy, etc.)

[1]Follow-up at 2-3 months for participants who were negative by eMRS (irrespective of index test). [2] Follow up at 6 months for those who were FujiLAM positive but were negative by composite reference standard. [3]Leftover urine samples will be collected and stored in FIND's biobank [4]If signs/symptoms are not improved or completely resolved

Specimen Flow

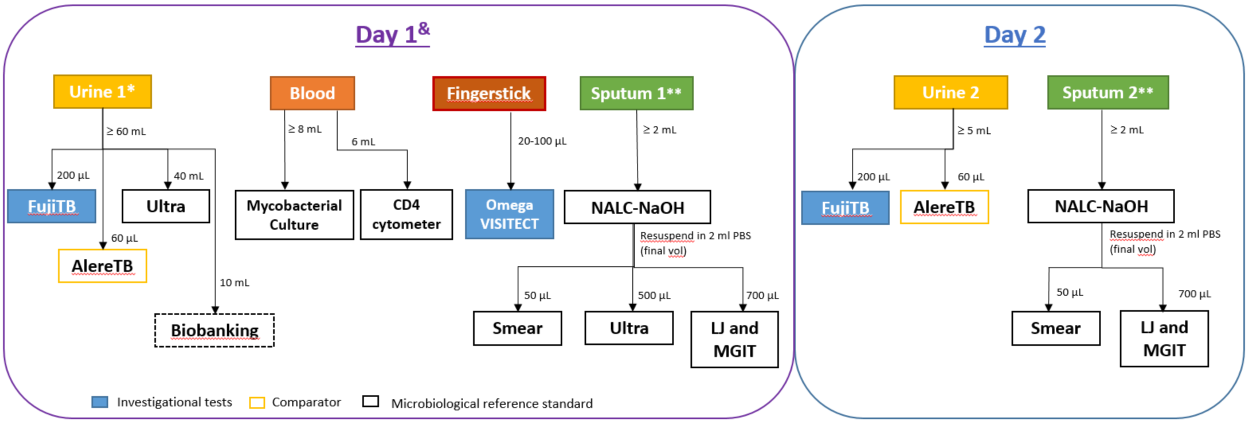

*Leftover urine (if min 7 ml) to be stored in the biobank

**For patients who are unable to provide expectorated sputum, an attempt will be made to obtain induced sputum.

& If direct sputum sample processing is required for routine submission, a second sputum sample should be collected. The first sputum sample should be used for trial purposes and the second for routine submission.

Note: All Day 1 specimens should be obtained the day of the medical admission (day of enrolment). Ideally, Day 2 specimen should be collected the following day of the Day1 urine collection but within maximum 7 days of enrolment as long as only ≤2 doses of anti-TB treatment were taken. All samples will be obtained wherever possible with reasonable efforts.

FIGURE 1 *SPECIMEN FLOW*

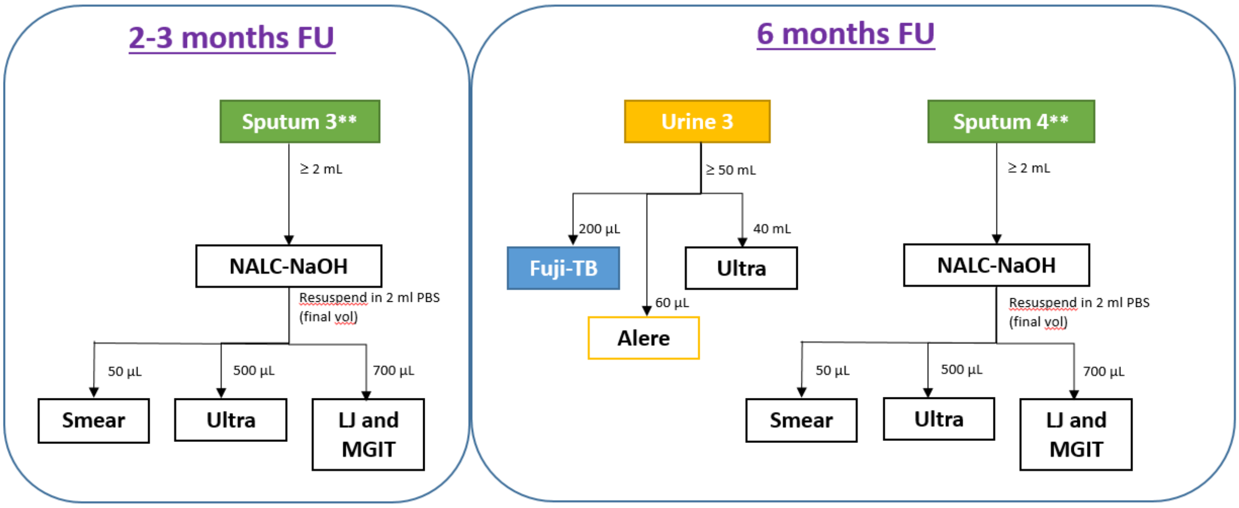

**For patients who are unable to provide expectorated sputum, an attempt will be made to obtain induced sputum.

Note: 2-3 months FU is required for those patients who are negative by eMRS (irrespective of index test). Follow up at 6 months is required for those who are FujiLAM positive but were negative by composite reference standard (CRS).

FIGURE 2 *SPECIMEN FLOW AT FOLLOW-UP.*

1 Introduction

Tuberculosis (TB) is the number one infectious disease killer. In 2016, 10.4 million fell ill with TB and an estimated 10% (range, 8–12%) of the incident TB cases were among people living with HIV[1]. TB is the most common cause of death in people living with HIV (PLHIV) and the risk of developing TB is estimated to be ~30 times greater in PLHIV than in people without HIV[2]. Most of the deaths from TB would be preventable if TB were diagnosed earlier; however, TB often goes undiagnosed.

1.1 Trial Rationale

Traditional diagnostic methods, such as culture or smear microscopy, are slow or low in sensitivity and more modern techniques, such as sequencing or Xpert® MTB/RIF, require specialized facilities, are costly, or are otherwise inaccessible to populations at greatest risk of contracting TB. Moreover, TB is harder to diagnose in PLHIV, since many of the patients have extrapulmonary TB (EPTB, ~25%) and paucibacillary TB, or may be too ill/weak to produce a sputum specimen. TB in PLHIV is almost certain to be fatal if undiagnosed or left untreated. New, rapid, non-sputum-based point-of-care (POC) diagnostic solutions to detect TB are urgently needed.

To address this unmet diagnostic need, Fujifilm and FIND have developed the biomarker-based Fujifilm SILVAMP TB LAM (FujiLAM) test. An initial study on frozen urine sample of inpatient PLHIV showed promising results but no data from prospective testing (of fresh urine) or from an outpatient population is available to date. The rationale of this trial is to fill this evidence gap.

FIND will continue to work on improvements to the assay to continue to increase diagnostic accuracy. Leftover urine specimens from the Day 1 collection (2 x 3.5 ml) will be stored at FIND's biorepository to assess new versions of FujiLAM compared to the original one.

In resource poor settings, many HIV positive patients do not have access to CD4+ T cells measurement to aid management of patients with pre-diagnosed HIV infection. Flow cytometry is the established reference method for CD4 testing and is often only available at centralised laboratories. Accurate, inexpensive POC tests are needed that can provide baseline CD4+ T cell estimates at the primary healthcare level. VISITECT® CD4 Advanced Disease is a rapid, affordable and instrument-free test that provides actionable results at point of care. Such a test will be of critical importance, if other tests for co-morbidities only have a recommendation for subsets of the PLHIV, i.e. AlereLAM is only recommended by the WHO currently for patients with CD4 <100 and an updated recommendation will likely expand the recommendation to patients with CD4 <200.

WHO recommends an advanced care package for patients with low CD4 count (cotrimoxazole prophylaxis, cryptococcal antigen screening, azithromycin prophylaxis), which is a challenge to community-based ART initiation. POC-CD4 is a promising tool for community-based HIV screening and care and may potentially also help increasing efficiency of TB screening.

1.2 Background

The FujiLAM test is based on the sensitive and specific detection of Lipoarabinomannan (LAM) in the urine of patients to diagnose active TB. The diagnostic sensitivity of FujiLAM relies on the use of two novel monoclonal antibodies with particular binding specificity to *M. tuberculosis* (MTB) LAM-epitopes present in TB patient's urine. The expected cut-off of the FujiLAM is ~30 pg/ml which is ~30-times below the cut-off of the marketed Alere Determine™ TB (AlereLAM) test.

A laboratory accuracy study on 968 previously collected frozen specimens was performed to assess the diagnostic performance of FujiLAM in comparison to that of AlereLAM for the detection of active TB in people living with HIV. TB was diagnosed in 600 participants (TB prevalence 62%) and the median CD4 count was 86 cells/µl. Using the MRS, point estimates of sensitivity of FujiLAM and AlereLAM were 70.4% (95%CI: 53.0–83.1) and 42.3% (95%CI: 53.0–83.1) respectively, while the specificity of FujiLAM and AlereLAM was 90.8% (86.0–94.4) and 95.0% (87.7–98.8) respectively. When using the CRS point estimates of sensitivity of FujiLAM and AlereLAM were 64.9% and 38.2% respectively, while the specificity of FujiLAM and AlereLAM was 95.7% and 98.2% respectively.[3]

This trial will also assess a novel rapid diagnostic test (RDT), called VISITECT® CD4 Advanced Disease test (<200 cells/µl).

The VISITECT® CD4 Advanced Disease count test is a lateral-flow POC test for the estimation of CD4 protein on the surface of CD4+ T cells in human whole blood to indicate whether the level is above or below 200 cells/µl.

The VISITECT® CD4 Advanced Disease *in vitro* diagnostic test might enable the introduction of algorithmic TB testing at the POC with LAM-based assays based on rapid stratification of PLHIV into those with less ≤200 or >200 CD4 count. The test results of CD4 ≤200 cells/µl also inform prophylaxis and cryptococcal screening.

The goal of this study is to assess the performance of both FujiLAM and VISITECT® CD4 Advanced Disease on prospectively collected, fresh specimens.

The data gathered from this trial, will form part of the dossier to be submitted to WHO for review.

While not directly related to the objectives of this study, FIND may opt to store the digital chest X-rays files (in DICOM format) collected during this study with the purpose of adding them to a databank used to design and validate artificial intelligence-based computer software for diagnostic and/or surveillance applications (e.g. detection of TB, like CAD4TB). The chest X-rays files will be completely anonymized before being stored, and would be linked to data that informed the microbiological and composite reference standards as well as the physician's interpretation of these X-rays. The data will be stored on a server owned and managed by FIND, which is located at FIND's premises in a secured, access-controlled room, and is not connected to any network except the FIND's internal one. This server is only used to store digital data for similar applications, and to run and evaluate the performance of such applications.

CONFIDENTIAL

1.3 Benefit/Risk Assessment

The FujiLAM and the VISITECT® CD4 Advanced Disease tests under investigation are considered low risk to trial participants, because required sample collection is considered to be non-invasive.

1.3.1. Potential risks associated with the study

Collection of spontaneously produced urine samples for FujiLAM from study participants is a non-invasive procedure with low risk. Minimal risks are associated with venipuncture for obtaining blood. Foreseeable adverse events associated with venepuncture are mild, temporary discomfort at the venepuncture site; bruising; and phlebitis. Very rarely, patients may experience vaso-vagal syndrome during phlebotomy. Vaso-vagal reactions may include diaphoresis, nausea, syncope, and rarely fainting. Minimal risks are associated with fingerstick sampling.

There are minimal risks associated with obtaining spontaneously expectorated sputum. Induced sputum, will only be done if clinically indicated. Adverse events associated with induced sputum collection are coughing, dry mouth, chest tightness, nausea and excess salivation. All other samples will be collected for routine care at the discretion of the clinical, non-study teams.

Handling and manipulation of pathogenic samples will be performed according to the country specific requirements to further minimize risk of contamination for the technical personnel and the environment. Given the nature of the study, termination due to safety or other reasons is not anticipated.

1.3.2. Known potential benefits

Knowledge gained from this study may benefit society by improving TB diagnosis in PLHIV in the future. Study participants may directly benefit from the study because they will be provided with a higher standard of TB diagnostic care than may be routinely available to them.

Given the minimal risks associated with this study and the potential benefits to society and individuals, the benefits outweigh the risks. As for any clinical study, there is a possibility of unknown and unforeseen risk; that possibility is small for this study. If unforeseen risks are recognized during the study, then FIND, study partners, IRBs/ethics committees, and participants will be provided with relevant information.

CONFIDENTIAL

2 Trial Objectives and Endpoints

OBJECTIVES	ENDPOINTS
**PRIMARY**	
1.1. To determine the diagnostic accuracy of FujiLAM for TB detection among PLHIV using an extended microbiological reference standard (eMRS)	1.1. Point estimates of sensitivity and specificity of FujiLAM, with 95% confidence intervals, using an eMRS
1.2. To determine the diagnostic accuracy of FujiLAM for TB detection among PLHIV using a composite reference standard (CRS)<br>1.3. To determine the diagnostic yield of FujiLAM, AlereLAM, Smear and Ultra among all patients identified by eMRS (on Day 1 specimens).	1.2. Point estimates of sensitivity and specificity of FujiLAM, with 95% confidence intervals, using a CRS<br>1.3. Diagnostic yield, with 95% confidence interval, of FujiLAM, AlereLAM, Smear and Ultra among eMRS-positive patients (on Day 1 specimens).
**SECONDARY**	
2.1. To determine the diagnostic accuracy of FujiLAM for TB detection among PLHIV using a microbiological reference standard (MRS)<br>2.2. To determine the diagnostic yield of FujiLAM, AlereLAM, Smear and Ultra (sputum, urine) among all patients identified by MRS (on Day 1 specimens).<br>2.3. To determine the diagnostic accuracy of FujiLAM across predefined subgroups* using a MRS, eMRS and CRS separately.<br>2.4. To determine the diagnostic accuracy of FujiLAM of Day 2 early morning urine using a MRS, eMRS and CRS separately.<br>2.5. To determine the diagnostic accuracy of FujiLAM of Day 2 early morning urine across subgroups using a MRS, eMRS and CRS separately.<br>2.6. To determine the diagnostic accuracy of AlereLAM in the same way as described in objectives 1.1, 1.2 and 2.1, 2.3 and estimate the difference to that of FujiLAM.<br>2.7. To determine the yield of FujiLAM over (incremental yield) or in combination with (combined yield) Smear and Ultra among eMRS-positive patients<br>2.8. To determine the association of positive FujiLAM results with mortality (only at sites where feasible)	2.1. Point estimates of sensitivity and specificity of FujiLAM, with 95% confidence intervals, using a MRS<br>2.2. Diagnostic yield, with 95% confidence interval, of FujiLAM, AlereLAM, Smear and Ultra among MRS-positive patients (on Day 1 specimens).<br>2.3. Point estimates of sensitivity and specificity of FujiLAM, with 95% confidence intervals, across predefined subgroups*, using a MRS, eMRS and CRS separately.<br>2.4. Point estimates of sensitivity and specificity, with 95% confidence intervals, of FujiLAM of Day 2 early morning urine, using a MRS, an eMRS and a CRS separately.<br>2.5. Point estimates of sensitivity and specificity, with 95% confidence intervals, of FujiLAM of Day 2 early morning urine across subgroups, using a MRS, an eMRS and a CRS separately.<br>2.6. Point estimates of sensitivity and specificity, with 95% confidence intervals, of AlereLAM in the same way as described in endpoints 1.1, 1.2 and 2.1, 2.3.<br>2.7. Yield of FujiLAM over (incremental yield) or in combination with (combined yield) Smear and Ultra among eMRS-positive patients.<br>2.8. Survival analysis for the outcome of death within 2-3 months by TB and FujiLAM status (only at sites where feasible)

CONFIDENTIAL

CONFIDENTIAL

2.9. To determine the diagnostic accuracy of VISITECT® CD4 Advanced Disease test for detection of a CD4 count ≤200 cells/µl, using WHO pre-qualified cytometers as the reference standard.<br>2.10. To assess the feasibility, ease of use of the FujiLAM test at POC	2.9. Point estimates of sensitivity and specificity, with 95% confidence intervals, of VISITECT® CD4 Advanced Disease test, using cytometry as the reference standard.<br>2.10. Time to proficiency, implementation issues, design related issues at POC

*CD4 strata defined by cytometer; CD4 strata defined by VISITECT® CD4 Advanced Disease; outpatient vs. inpatients; by ART status; PTB vs EPTB vs PTB&EPTB; WHO danger signs (respiratory rate > 30/min, heart rate > 120/min, temperature > 39 °C, and being unable to walk unaided); number of anatomical sites compromised; WHO clinical stage of HIV disease; TB history; enrolment sites

3 Trial Design

3.1 General Design

This is a prospective, multicentre cohort study in which the accuracy and the diagnostic yield of FujiLAM test will be assessed using a comprehensive microbiological reference standard (MRS), an extended MRS (eMRS) and composite reference standard (CRS). Definitions of MRS, eMRS and CRS are under Section 6.2.1.

Adult PLHIV (≥ 18 years of age), will be screened for eligibility according to the inclusion and exclusion criteria (Section 3.5).

Urine, respiratory and blood specimens will be collected and processed fresh from participants after informed consent is obtained.

In order to assess the impact of early morning urine collection to FujiLAM performance a second urine will be collected on Day 2[4].

3.2 Scientific Rationale for Trial Design

In order for the results of this trial to be generalizable, adult PLHIV will be screened for inclusion at 6 geographically diverse participating centres in high burden HIV/TB countries. Following the assessment of the performance in a laboratory accuracy study on frozen specimens, this will be the first large scale evaluation on prospectively collected fresh samples.

Imperfect mycobacterial reference standard can lead to bias in the accuracy estimates of the index test therefore, the accuracy of FujiLAM will be calculated using multiple reference standards such as MRS, eMRS and CRS as defined at the beginning of the protocol. The cohort design with predefined follow-up period will overcome the challenges of using MRS only.

To evaluate the possible clinical effectiveness of the index test, the diagnostic yield and the mortality will be assessed.

In order to achieve a broad spread of data on FujiLAM across CD4 strata, the optimal distribution of the PLHIV would be 50% of inpatients and 50% of outpatients.

CONFIDENTIAL

3.3 End of Trial Definition

The end of the study is defined as the date of the last visit as per the Schedule of Activities for the last participant in the study globally or as the date when all the results of his/her microbiological assessments are reported (including follow-up), whichever comes last.

3.4 Trial Population and Eligibility

- Trial population: adult PLHIV (≥18 years), irrespective of their CD4 count and antiretroviral therapy (ART) status, at risk of having pulmonary and/or extra-pulmonary TB
- Location: the trial will take place in 6 different countries:
  - 5 sites in sub-Saharan Africa
    - Malawi
    - South Africa
    - Tanzania
    - Uganda
    - Zambia
  - 1 site in Asia
    - Vietnam

Setting: participants will be recruited at HIV outpatient and inpatient settings.

Prospective approval of protocol deviations to recruitment and enrolment criteria, also known as protocol waivers or exemptions, is not permitted.

3.5 Inclusion Criteria

Patients are eligible to be included as trial participants only if all of the following inclusion criteria apply:

- HIV positive (self-record or documented), irrespective of CD4 cell count and ART treatment.
- Aged ≥18 years.
- TB symptoms, depending on the settings:
  - Outpatients → At least one of the symptoms below suggestive of TB (as defined by WHO)
    - Current cough
    - Night sweats
    - Fever
    - Weight loss
  - Inpatients → Irrespective of TB symptoms, those requiring acute admission to medical wards
- Provision of written informed consent.
- Willingness to have a trial follow-up visit at 2-3 and 6 months after enrolment (e.g. not planning to relocate).

3.6 Exclusion Criteria

Participants are excluded from the trial if any of the following exclusion criteria apply:

- Current anti-TB treatment *
- Anti-TB treatment within 60 days prior to enrolment
- Receipt of isoniazid preventive therapy within 6 months prior to enrolment.

*Note: Patients starting anti-TB treatment at the time of enrolment will not be excluded from the trial provided that all trial specimens are collected before starting the 3rd dose of treatment.

3.7 Early exclusions

No early exclusions in this trial.

4 Trial Intervention

Trial Intervention is defined as any investigational intervention(s), marketed product(s), or medical device(s) intended to be used with a trial participant according to the trial protocol. Results of investigational tests (FujiLAM and VISITECT® CD4 Advanced Disease) will not be used for patient management. Results of the AlereLAM tests can only be used for patient management if the test is approved in the country.

4.1 Investigational Product

4.1.1 Investigational Product: Fujifilm SILVAMP TB LAM (FujiLAM) test

FujiLAM is a visually-read, qualitative, rapid, in-vitro diagnostic test for the sensitive detection of lipoarabinomannan (LAM) antigen of MTB in human urine as an aid in the diagnosis of active TB. The cartridge and biological principle are shown in the **Figure 3**.

FujiLAM employs two monoclonal antibodies which bind to glycan capping motifs of LAM that are unique to slow-growing mycobacteria. A urine specimen is pre-incubated with the gold-nanoparticle conjugated detection antibody in the reagent tube. After the pre-incubation, urine is added to the sample port (1) and the LAM-antibody-gold particle complex travels to the test line where the capture antibody, which is immobilized on the nitrocellulose membrane, binds the complex. The test user then releases a reducing reagent by pressing (2) and a silver ion liquid by pressing (3). This results in the formation of silver clusters of 5-10 µm diameter around the gold particles and a black test line. This Silver Amplification Immunochromatography (SAI) enables a 30-fold lower cut-off compared to conventional lateral flow immunoassays and results in positive test line if a sample includes concentrations ≥30 pg LAM/ml urine. To ensure assay validity, a procedural control line is incorporated in the test cartridge.

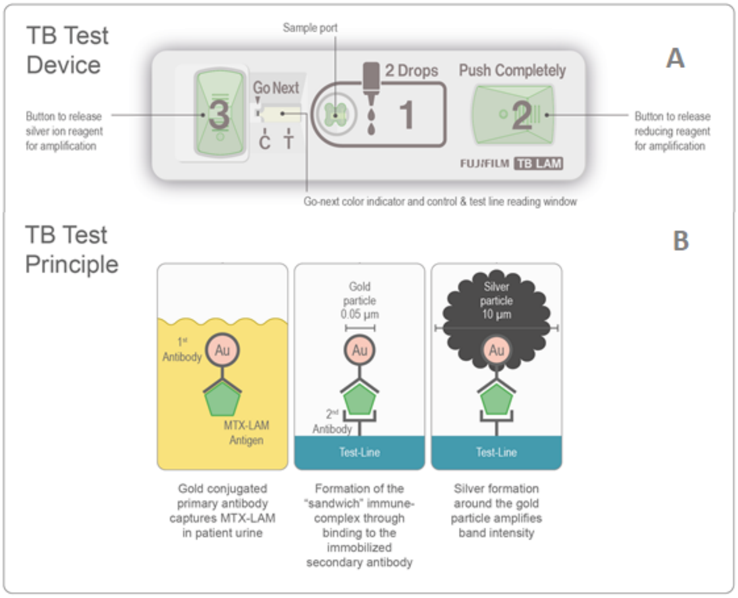

FIGURE 3: FUJILAM TEST. (A) CARTRIDGE AND (B) BIOLOGICAL PRINCIPLE: URINE SPECIMEN IS PRE-INCUBATED WITH THE GOLD-NANOPARTICLE CONJUGATED DETECTION ANTIBODY AND ADDED TO THE SAMPLE PORT (1). THE LAM-ANTIBODY-GOLD PARTICLE COMPLEX TRAVELS TO THE TEST LINE WHERE THE IMMOBILIZED CAPTURE ANTIBODY BINDS THE COMPLEX. AFTER RELEASING THE REDUCING REAGENT BY PRESSING (2) AND A SILVER ION LIQUID BY PRESSING (3) THE CONTROL AND, IN THE POSITIVE CASE, THE TEST LINE BECOME VISIBLE.

4.1.2 Comparator: Alere Determine™ TB LAM (AlereLAM)

The AlereLAM is a commercially available lateral flow assay that detects lipoarabinomannan, a lipopolysaccharide present in mycobacterial cell walls. It is currently recommended by the WHO for point-of-care testing for the diagnosis of active TB in PLHIV with CD4≤100 cells/µl and in those who meet the WHO-defined criteria of being 'seriously ill'. AlereLAM will soon be reviewed for use in PLHIV with CD4 <200 cells/µl by the WHO based on novel data. The AlereLAM will be performed as a comparator according to the test's package insert.

Moreover, for objective 1.3 (Day 1 diagnostic yield) Ultra (sputum, urine) and smear will also be used as comparators.

4.1.3 Investigational product: VISITECT® CD4 Advanced Disease Rapid Test

The VISITECT® CD4 Advanced Disease Rapid Test is an immunochromatographic assay that estimates full length CD4 protein associated with CD4+ T-cells in human whole blood and is directly correlated with CD4+ T-cell levels.

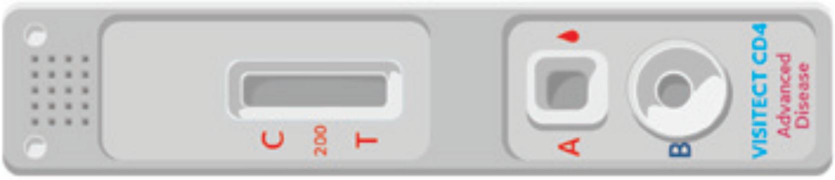

FIGURE 4: VISITECT® CD4 ADVANCED DISEASE TEST (<200 CELLS/µL). RESULTS WINDOW WITH CONTROL LINE (C), REFERENCE LINE (200) AND TEST LINE; A=WELL FOR BLOOD SAMPLE; B=WELL FOR BUFFER

A capture monoclonal antibody (MAb) specific for the cytoplasmic domain of CD4 is applied as a line on the nitrocellulose membrane. Whole blood is added directly to the VISITECT® CD4 Advanced Disease Rapid Test where red blood cells and monocytes are retained in the blood collection pad and following the addition of buffer, other white blood cells (including CD4+ T-cells) migrate to a reaction area where cell lysis occurs, resulting in the release of full-length CD4 for capture in the test strip. Colloidal gold labelled MAb conjugate against CD4 binds the captured CD4 and forms a test line. These complexes are visualised as a pink/purple line. A reference line (200 line) is included to allow estimation of CD4 levels by comparison to a set cut-off point (e.g. equivalent to the signal level generated by samples containing 200 CD4+ T-cells/µL). The 200 line and control line must be present for the assay result to be valid. The control line in the test device is not a sample addition control.

4.2 Preparation/Handling/Storage/Accountability

Acquisition

Procurement of the investigational products will be done through FIND, who will coordinate shipments from the manufacturer. It is the responsibility of each trial site to maintain an updated inventory of the trial materials and to inform FIND immediately if additional materials are required.

The investigator or designee must confirm appropriate temperature conditions have been maintained during transit for the investigational product received and any discrepancies are reported and resolved before its use.

Storage

Procedures for product storage and disposal will be described in the Trial Manual[i].

The investigational product must be stored in a secure, environmentally controlled, and monitored (manual or automated) area in accordance with the labelled storage conditions with access limited to the investigator and authorized site staff.

Test Handling and Performance

Testing using the investigational products will be performed according to the manufacturer's instructions outlined within the Trial Manual.

Only urine samples from participants enrolled in the trial will be processed with the FujiLAM investigational product and only authorized site staff will be responsible for processing.

Only fingerstick blood samples from participants enrolled in the trial will be processed with the VISITECT® CD4 Advanced Disease investigational product and only authorized site staff will be responsible for processing.

[i] The Trial Manual will comprise all trial-specific Standard Operating Procedures, Work Instructions, Tools and Templates.

Accountability

The investigator is responsible for trial intervention accountability, reconciliation, and record maintenance (i.e., receipt, reconciliation, and final disposition records). Investigational Product Accountability logs filled at each site will ensure the proper follow-up of the used, failed and remaining investigational products

Further guidance and information for the final disposition of unused investigational product are provided in the Trial Manual.

Export and Import Permits

It is expected that most countries will require import permits for receiving the investigational materials. Local sites are responsible for making import permit applications in a timely manner.

Quality Control Check for Incoming Shipments

Upon arrival of each new shipment of assays, the sites will conduct and document an incoming quality check following the Trial Manual. New lots may only be used after this quality check is successfully passed.

Local procurement

Sites are responsible for assessing their needs and procuring any supplies, reagents and kits needed for the trial that are locally available in order to include these costs in the trial budget.

4.3 Minimisation of Error and Bias

4.3.1. Patient selection

Spectrum bias will be avoided by enrolling a consecutive series of study participants, and by using a prospective trial design. Enrolment will be based on clearly defined eligibility criteria. The most severely ill patients cannot be enrolled, as drowsy or confused inpatients cannot provide informed consent and this may lead to a degree of underestimation of sensitivity. However, to ensure the validity and generalizability of study results, descriptive statistics on patient characteristics and estimates of diagnostic accuracy will be reported separately for relevant subgroups (outpatients/inpatients, CD4 cell count, WHO clinical stage of HIV disease, number of anatomical sites diseased with TB, WHO danger signs etc.) that are a proxy for disease spectrum/severity.
To avoid bias originating from the nature of the narrow participant population and *Mycobacterium tuberculosis* lineage a multicentre study will be performed on different geographic areas.

4.3.2. Index test

The overall risk of review bias is minimal as the FujiLAM threshold is pre-defined and since lab personnel interpreting results will be blinded to all other test results. In order to ensure blinding of FujiLAM results to the AlereLAM comparator and to the result of the microbiological reference standard including Ultra, different operators will be assigned and will be instructed to record results independently of other test results.

Harmonization of result interpretation will be ensured with proper training, proficiency assessment and competency assessment done on a regular basis during the trial period.

4.3.3. Reference standard and comparator tests

Each participating laboratory will undergo an on-site laboratory evaluation to ensure standardized and high-quality performance of culture (sputum, blood), smear and Ultra (sputum, urine). Results from reference standard testing are either generated automatically or will be recorded blinded to the index test and comparator test results, eliminating the risk of review bias.
The MRS has imperfect sensitivity, which can lead to bias in the accuracy estimates of the index test: sensitivity will be overestimated when index test and reference standard both yield negative results in a patient with TB, and specificity will be underestimated when the index test gives a positive result in a patient with TB who tests negative with the MRS. Therefore, the accuracy of FujiLAM will be calculated using MRS, eMRS and CRS separately. The possibility of "false"-positive FujiLAM results has been described and attributed to a likely misclassification by the reference standard[3]. In order to address this, extensive TB testing will be performed including patient follow-up after 2-3 and 6 months (Section 6.1, Table 1).

4.3.4. Flow and timing

Samples for index test testing will be collected in parallel to the samples that will be used for reference testing so disease progression bias is not a concern.

5 Participant Discontinuation/Withdrawal

5.1 Participant Discontinuation/Withdrawal from the Trial

A participant may be withdrawn at any time at the discretion of the investigator for safety, behavioural, compliance, or administrative reasons.

If the participant withdraws consent for disclosure of future information, FIND may retain and continue to use any data collected before such a withdrawal of consent.

If a participant withdraws from the trial, he/she may request destruction of any samples taken and not tested, and the investigator must document this in the site trial records.

See Schedule of Activities for data and/or samples to be collected at the time of trial discontinuation and follow-up and for any further evaluations that need to be completed.

5.2 Lost to Follow Up

A participant will be considered lost to follow-up if he or she repeatedly fails to return for scheduled visits and is unable to be contacted by the trial site. Should the participant continue to be unreachable after 3 attempts within 4 weeks of the due follow-up date, the participant will be considered to be lost to follow-up.

The following actions must be taken if a participant fails to return to the clinic for a required trial visit:

- The site must attempt to contact the participant and reschedule the missed visit as soon as possible and counsel the participant on the importance of maintaining the assigned visit schedule and ascertain whether or not the participant wishes to and/or should continue in the trial.
- Before a participant is deemed lost to follow up, the investigator or designee must make every effort to regain contact with the participant. These contact attempts should be documented in the participant's study record.
- Should the participant continue to be unreachable, he/she will be considered to have withdrawn from the trial.

CONFIDENTIAL

6 Trial Procedures

Trial procedures and their timing are summarized in the Schedule of Activities. PLHIV, compliant with the eligibility criteria, will be prospectively recruited at outpatient clinic settings and inpatient hospital settings.

Outpatients with at least one symptom of the WHO symptom screen suggestive of TB (current cough, night sweats, fever, weight loss) and inpatients irrespective of TB symptoms will be asked by non-trial clinicians or appropriately trained staff if they would be interested in participating in the trial. Interested individuals will be referred to trial personnel for additional information and screening. The investigator will maintain a screening log to record details of all participants screened and to confirm eligibility or record reasons for screening failure, as applicable.

Following the informed consent process samples will be obtained. Demographic and health related information including signs/symptoms and TB history will also be collected.

Procedures conducted as part of the participant's routine clinical management (e.g., CXR, HIV testing) and obtained before signing of the ICF may be utilized for screening or baseline purposes provided the procedures met the protocol-specified criteria and were performed within the time frame defined in the Schedule of Activities if applicable.

Adherence to the trial design requirements, including those specified in the Schedule of Activities, is essential and required for trial conduct.

6.1 Specimen Collection, Handling, Storage

Specimen collection, handling, transport and storage will be carried out according to local policies and to instructions provided in the Trial Manual (for non-routine procedures).

Trial sites will make sure that all measures are in place to make systematic sample collection possible.

<u>Testing at enrolment</u>

Day 1

Eligible and consented adults, meeting all of the inclusion criteria and none of the exclusion criteria, will be asked to provide urine, blood and respiratory specimens on the first day of medical admission or during their outpatient visit. Specimens will be obtained as outlined in the schedule of activities and testing will be done as shown in the specimen flow (Figure 5).

In brief, on Day 1 the following samples and quantities will be collected:

- ≥ 60 ml urine for FujiLAM, AlereLAM, large-volume (i.e. 30ml) Ultra testing, 10 ml urine for biobanking and discordant analysis
- ≥ 8 ml blood for blood culture
- 6 ml blood for laboratory-based CD4 cell count determination
- Fingerstick (30 µl) for:
    - VISITECT® CD4 Advanced Disease rapid diagnostic test
- 2-3 ml sputum (decontaminated) for smear microscopy, Ultra, LJ and MGIT culture

Note: If direct sputum sample processing is required for routine testing, a second sputum sample should be collected the same day.

The first sputum sample should be used for trial purposes and the second for routine testing.

Participants will also be sent for chest X-ray examination unless already obtained as part of routine clinical management. Once identifiable details are removed, these chest X-rays may be used by FIND for storage in a databank for artificial intelligence-based computer programs, as highlighted above.

Day 2

The following samples should be collected within 7 days of enrolment as long as only ≤2 doses of anti-TB treatment were taken:

- ≥ 5ml early morning urine for FujiLAM, AlereLAM

  Outpatients can collect early morning urine at home and bring it back to the clinic.

- ≥ 2 ml sputum (decontaminated) for smear microscopy, LJ and MGIT culture

For patients who are unable to provide expectorated sputum, an attempt will be made to obtain induced sputum if considered clinically indicated.

CONFIDENTIAL

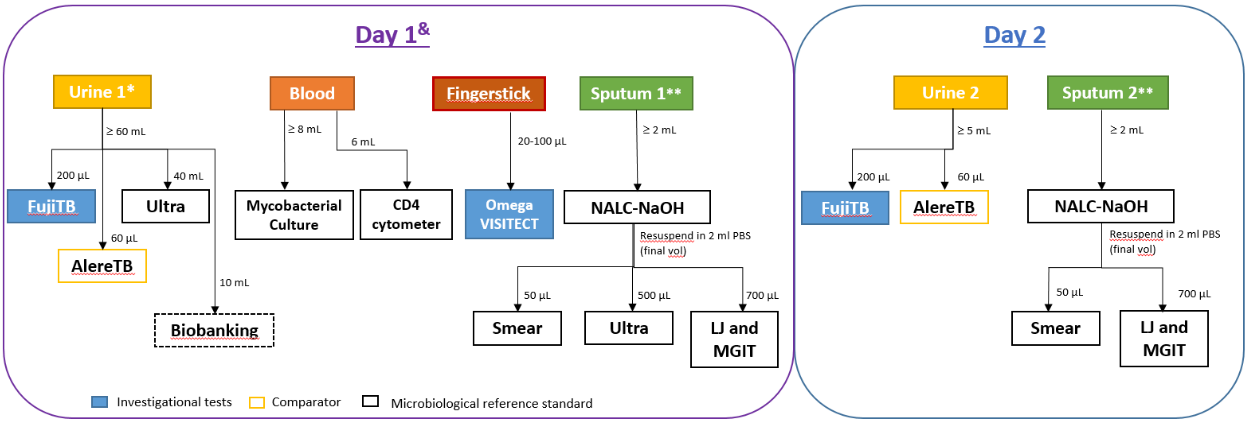

*Leftover urine (if min 7 ml) to be stored in the biobank
**For patients who are unable to provide expectorated sputum, an attempt will be made to obtain induced sputum.
& If direct sputum sample processing is required for routine submission, a second sputum sample should be collected. The first sputum sample should be used for trial purposes and the second for routine submission.

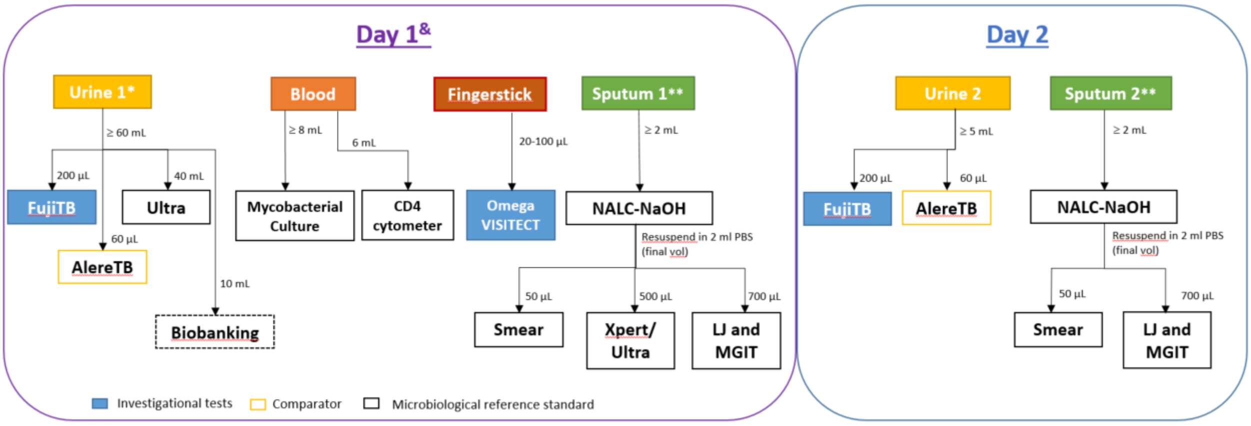

*Leftover urine (if min 7 ml) to be stored in the biobank
**For patients who are unable to provide expectorated sputum, an attempt will be made to obtain induced sputum.
& If direct sputum sample processing is required for routine submission, a second sputum sample should be collected. The first sputum sample should be used for trial purposes and the second for routine submission.

***Figure 5** Specimen Flow*

Follow-up testing

Patients with negative eMRS will be asked to come back for a follow-up visit after 2-3 months of enrolment. An additional sputum specimen will be requested if all the criteria mentioned in Table 1 apply.

Patients with positive FujiLAM result and negative CRS will be asked to come back for an additional follow-up visit after 6 months of enrolment. An additional sputum and urine specimen will be requested if all the following criteria mentioned in Table 1 apply.

	Indication for sample collection at FU visits	
	**2-3 months FU**	**6 months FU**
**Inpatients**	• negative on eMRS **AND**	• negative on CRS **AND**

CONFIDENTIAL

	• not started on TB treatment	• positive on FujiLAM
**Outpatients**	• negative on eMRS<br>**AND**<br>• not started on TB treatment<br>**AND**<br>• signs and symptoms of TB are persistent	• negative on CRS<br>**AND**<br>• positive on FujiLAM<br>**AND**<br>• signs and symptoms of TB are persistent

TABLE 1 INDICATION FOR SAMPLE COLLECTION AT FU VISITS

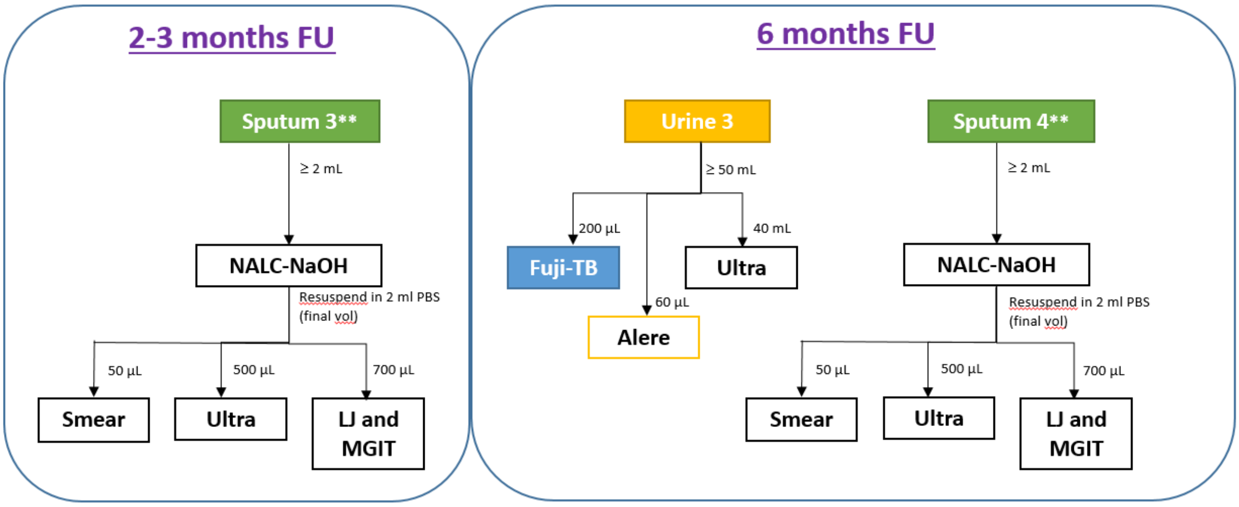

**For patients who are unable to provide expectorated sputum, an attempt will be made to obtain induced sputum.

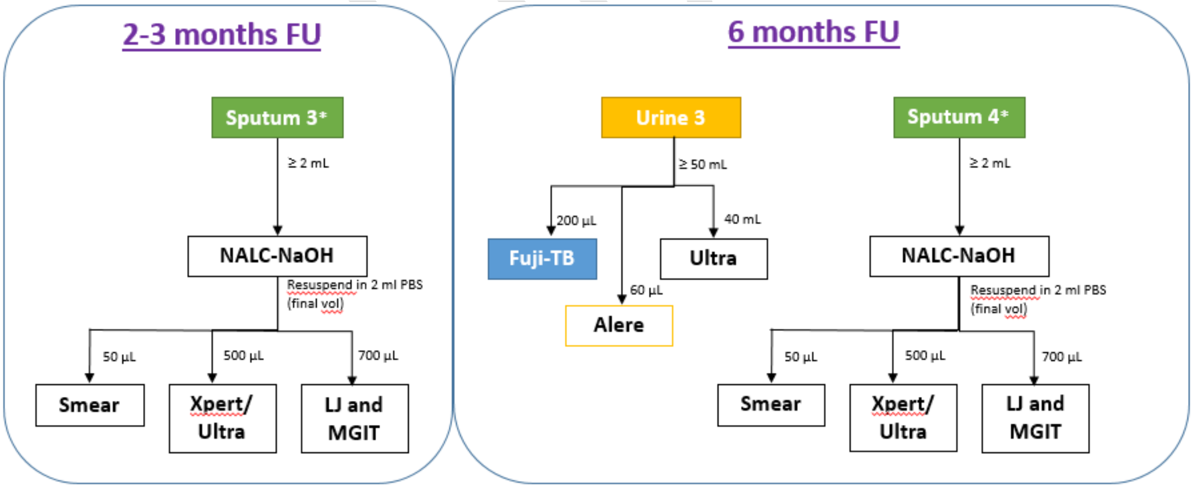

*For patients who are unable to provide expectorated sputum, an attempt will be made to obtain induced sputum.

FIGURE 6 SPECIMEN FLOW AT FOLLOW-UP

Additional testing

Furthermore, results from additional mycobacterial culture or Xpert or Ultra from other respiratory and/or non-respiratory samples (e.g. pleural fluid, tissue biopsy, etc.) done as part of routine clinical management will be recorded during the entire trial period.

Storage

Leftover respiratory samples (decontaminated sputum pellet) and a small aliquot of urine (1 ml) will be kept frozen until the end of the trial and data analysis is completed.

FIND continues to work on improvements of the assay. Leftover urine specimen from the Day 1 collection will be stored at FIND's biorepository (2 x 3.5 ml) to assess new versions of FujiLAM compared to the original one.

Maintaining adequate storage conditions will be crucial for the quality of reference materials. Urine samples must be stored on site at -70°C to -80°C immediately after aliquoting. A convenient storage system using boxes with individual holders and storage templates should be implemented to facilitate easy identification and location of the aliquots. Storage templates will be provided in the Study Manual.

A freezer alarm system and back-up power supply should be available. Temperature fluctuations and thaw-freeze of specimens can destroy valuable specimen characteristics and should therefore be avoided. Each thaw-freeze cycle undergone by the specimens or any exposure to temperatures considerably higher than -70°C, must be recorded and reported to FIND as an incident.

Urine samples collected in this study for biobanking purposes will be stored at FIND's repository for up to 20 years after the project end, an extension of this duration may be granted from the relevant ethics committees.

Study participants can request that their samples to be destroyed at any time point during or after the study, should they decide to withdraw from the study.

The necessary Material Transfer Agreements (MTA) will be signed prior to sample transport if not requested earlier by the sites.

Details regarding the transport, packaging and documentation will be described in the Study Manual. Sites are responsible for applying for the necessary export permits to allow shipment of the banked specimens to FIND repository. In order to avoid delays due to lengthy export permit applications, sites should apply for export permits immediately after study approval.

6.2 Reference Standard Test and Investigational Test Procedures

6.2.1. Reference Standard Tests for TB diagnosis

The list of tests considered for each and the definition of the different reference standards is shown below.

Smear microscopy, Ultra, LJ and MGIT culture on sputum samples as well as blood culture will be performed at the testing sites based on local SOPs for routine testing. Ultra on urine samples will be performed as described in the Trial Manual.

Results from reference standard testing will be recorded blinded to the index test results.

	**MRS**	**eMRS**	**CRS***
1-2 Sputum MGIT culture[Ω]	X	X	X
1-2 Sputum LJ culture[Ω]	X	X	X
Blood culture[Ω]	X	X	X
Urine Ultra	X	X	X
Sputum Ultra	X	X	X
AlereLAM[¥]			X
Additional (non-study) testing[§]		X	X
2-3 months testing	X	X	X
Anti-TB therapy			X
Reference standard **positive** if	Any of the MRS tests is positive	Any of the eMRS tests is positive	Any of the eMRS tests is positive and/or TB treatment was started, and response to treatment with follow-up is documented
Reference standard **negative** if	None of the MRS tests is positive and at least one sputum culture is negative	None of the eMRS tests is positive and at least one sputum culture is negative	None of the eMRS tests is positive and TB treatment was not started and the patient has no symptoms at 2-3 months
Unclassifiable	Neither Reference standard **positive** **nor** Reference standard **negative**		

Table 2 List of tests and Definitions of Reference Standards

MRS= microbiological reference standard; eMRS=extended MRS; CRS=composite reference standard
Ω Including MTB complex confirmation and NTM determination
§ Any additional mycobacterial culture and/or Xpert/Ultra from other samples (e.g. pleural fluid, tissue biopsy, etc.) performed based on routine clinical indication.
*Chest X-Ray might be considered as part of the clinical decision making.
¥If the test is used in the country for clinical decision making.

NOTE: Given that cases that are smear positive but Ultra or culture negative, likely are FP (either due to other mycobacteria or due to pre-treatment), we prefer to not include smear in MRS. Those cases will be reflected in CRS if physician makes a decision based on smear result.

6.2.2. FujiLAM Investigational Test Procedure

Processing of the FujiLAM index test will be performed on urine samples collected on Day 1, Day 2 and 6 months FU (if applicable) based on the instructions for use provided by the assay manufacturer and described in the Trial Manual.

Briefly, the 5-step test procedure is as follows: approximately 200 µl of urine are added to the reagent tube (up to the indicator line), mixed and incubated for 40 minutes at ambient temperature. After mixing the contents of the reagent tube again, two drops are added to the test strip (position “1”). Following this, button “2” is immediately pressed to release a reducing agent for silver amplification. After the “go-next” colour indicator mark turns orange (within approximately 3-10 minutes), button “3” is pressed to release a silver ion solution to activate the silver amplification reaction and the result is ready within 10 minutes. Test results will be visually interpreted in a blinded fashion (i.e. persons will not have access to other results from reference standard tests, the index test and to other comparator test results).

Results from the FujiLAM test will not be communicated to the clinical team, and measures will be taken to ensure that they do not influence clinical decision-making.

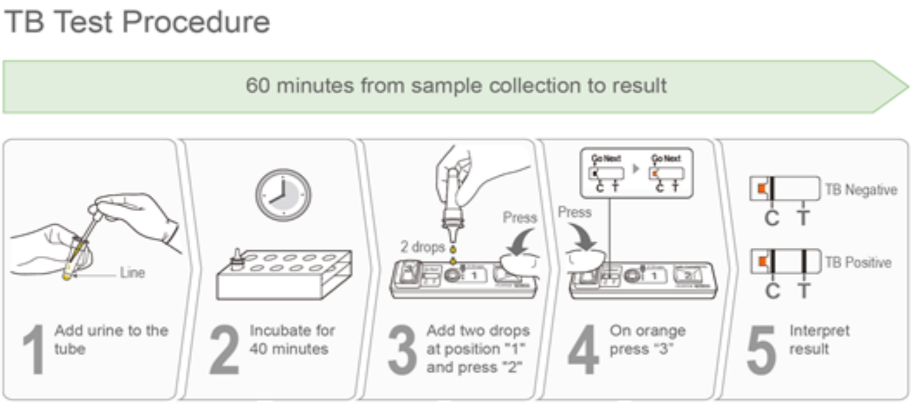

FIGURE 7: 5-STEP TEST PROCEDURE

6.2.3. VISITECT® CD4 Advanced Disease test (≤200 cells/µl)

Processing of the Omega test will be performed on venous blood obtained by fingerstick on Day 1, based on the instructions for use provided by the assay manufacturer and described in the Trial Manual.

Briefly, 30 µl of fingerstick blood will be added by a sampling device directly to the Well A. After 3 minutes of waiting time, 1 drop of buffer should be added to the same Well A. After 17 minutes, 3 drops of buffer should be added to Well B. Results can be interpreted after 20 minutes.

Test results will be visually interpreted in singlicate. A reference line (200 line) helps to allow estimation of CD4 levels by comparison to a set cut-off point (e.g. equivalent to the signal level generated by samples containing 200 CD4+ T-cells/µL). The 200 line and control line must be present for the assay result to be valid. Invalid tests results should be retested using the same sample in singlicate. If the same sample is no longer available then additional samples must be substituted. The invalid results should be recorded on the results sheet to enable an estimation of the invalid test rate.

Flow cytometers, prequalified by WHO, will be used as a reference standard for the VISITECT® CD4 Advanced Disease.

6.3 Comparator test procedure

AlereLAM

The AlereLAM will be performed on urine samples collected on Day 1, Day 2 and 6 months FU (if applicable) according to package insert.

The AlereLAM will be interpreted visually, using the reference card, by the operator in a blinded fashion (i.e. persons will not have access to other results from reference standard tests, the index test and to other comparator test results).

6.4 Safety Assessments

Given the nature of the trial, participant safety assessments will only include deaths. Refer to Section 7 Safety and Incident Reporting.

Laboratory safety assessments will be done during trial site assessment and/or trial initiation visits to ensure that potentially infectious specimens are processed according to national and/or international guidelines.

Medical device incidents will be recorded and reported according to the MEDDEV 2.12/1 Rev 8 (see summary in 7.1.3 Medical Device Incidents (including Malfunctions)).

7 Safety and Incident Reporting

Given that this is a diagnostic accuracy trial that is not utilizing test results for patient care and that additional procedures for the trial (i.e. sputum collection, venepuncture and fingerstick are extremely low risk), the probability of an AE or SAE occurring to a trial participant to be associated with the investigational products is extremely low.

CONFIDENTIAL

7.1 Adverse Events and Serious Adverse Events

The definitions of an Adverse Event (AE) and Serious Adverse Event (SAE) are considered as per MEDDEV 2.7/1 Rev 4. A brief summary can be found in Appendix 1.

Given the nature of this trial AE reporting is limited in scope to:

- SAEs that may be associated with sputum, urine or blood collection.
- SAEs that occur at the testing sites (reference laboratories) using the investigational product (see section 7.1.3 Medical device incidents).
- Any other serious events that affect the rights safety or welfare of subjects.

7.1.1 Time Period for Collecting SAE Information

Information will be collected from the first specimen collection until the follow-up visit at the time points specified in the Schedule of Activities. SAEs will be recorded and reported to the sponsor or designee within 24 hours of the occurrence, as indicated in Appendix 1. The investigator will submit any updated SAE data to the sponsor within 24 hours of being made aware of the event. The method of recording, evaluating and assessing causality of the SAE and the procedures for completing and transmitting SAE reports are provided in Appendix 1.

Investigators are not obligated to actively seek SAEs after conclusion of the trial participation. However, if the investigator learns of any SAE, including a death, at any time after a participant has been discharged from the trial, and he/she considers the event to be reasonably related to the trial intervention or trial participation, the investigator must promptly notify FIND.

7.1.2 Reporting and Follow up of SAEs

Prompt notification by the investigator to FIND of a SAE is essential so that legal obligations and ethical responsibilities towards the safety of participants and the safety of a Trial intervention under clinical investigation are met.
FIND has a legal responsibility to notify both the local regulatory authority and other regulatory agencies about the safety of a trial intervention under clinical investigation. FIND will comply with country-specific regulatory requirements relating to safety reporting to the regulatory authority, Institutional Review Boards (IRB)/Independent Ethics Committees (IEC), and investigators.
An investigator who receives a safety report describing a SAE or other specific safety information (e.g., summary or listing of SAEs) from FIND will review and then file it in the Investigator Site File (ISF) and will notify the IRB/IEC, if appropriate according to local requirements.

7.1.3 Medical Device Incidents (including Malfunctions)

Medical devices are being provided for use in this Trial for TB diagnosis in PLHIV. In order to fulfil regulatory reporting obligations worldwide, the investigator is responsible for the detection and documentation of events meeting the definitions of incident or malfunction that occur during the trial with such devices.

The definition of a Medical Device Incident can be found in Appendix 2.

NOTE: Incidents fulfilling the definition of an SAE will also follow the processes outlined above and in Appendix 1 of the protocol.

7.1.4 Time Period for Detecting Medical Device Incidents

Medical device incidents or malfunctions of the device that result in an incident will be detected, documented, and reported during all periods of the trial in which the medical device is used.
If the investigator learns of any incident at any time after a participant has been discharged from the trial, and such incident is considered reasonably related to a medical device provided for the trial, the investigator will promptly notify FIND.
The method of documenting Medical Device Incidents is provided in Appendix 2.

7.1.5 Follow-up of Medical Device Incidents

All medical device incidents involving an SAE will be followed and reported in the same manner as other SAEs (see Section 7.1.2). This applies to all participants, including those who discontinue trial intervention.

The investigator is responsible for ensuring that follow-up includes any supplemental investigations as indicated to elucidate the nature and/or causality of the incident.
New or updated information will be recorded on the originally completed form with all changes signed and dated by the investigator.

7.1.6 Reporting of Medical Device Incidents to FIND

Medical device incidents will be reported to FIND within 24 hours after the investigator determines that the event meets the protocol definition of a medical device incident.
The Medical Device Incident Report Form will be sent to the sponsor scanned by e-mail. If e-mail is unavailable, then the investigator should phone the sponsor and send an e-mail as soon as possible. Contact details will be provided in the Trial Manual.
The same individual will be the contact for the receipt of medical device reports and SAE.
Medical device incidents will then be communicated by the sponsor to the manufacturer within 24 hours after the sponsor has received the Medical Device Incident Report Form.

7.1.7 Regulatory Reporting Requirements for Medical Device Incidents

The investigator will promptly report all incidents occurring with any medical device provided for use in the Trial in order for FIND to fulfil the legal responsibility to notify appropriate regulatory authorities and other entities about certain safety information relating to medical devices being used in clinical studies.

CONFIDENTIAL

The investigator, or responsible person according to local requirements (e.g., the head of the medical institution), will comply with the applicable local regulatory requirements relating to the reporting of incidents to the IRB/IEC.

8 Statistical Considerations

The statistical analysis plan (SAP) will be developed and finalized before the start of enrolment; this section details a summary of the planned statistical analyses of the primary and secondary endpoints, as well as the rationale for the proposed sample size.

8.1 Populations for Analyses

For purposes of analysis, the following populations are defined:

TABLE 3 POPULATION FOR ANALYSES

Population	Description
Enrolled/Intention-to-test (ITT)	All subjects successfully enrolled in the study (having signed the ICF)
Evaluable/Per Protocol Population (PP)	All subjects in ITT who have urine samples available, and valid results for all tests
Survival population	All subjects in ITT who have data on vital status at 2-3 month FU

8.2 Statistical Analyses

<u>General Methodology</u>

Point estimates of sensitivity and specificity, with 95% confidence intervals based on Wilson's score methods, will be calculated following the definitions in **Error! Reference source not found.** and **Error! Reference source not found.**.

TABLE 4 CONFUSION MATRIX: DEFINITION OF TEST RESULTS

	Reference standard classification			
**Case prediction**		Positive	Negative	Total
	Predicted positive	a	b	(a + b)
	Predicted negative	c	d	(c + d)
	Total	(a + c)	(b + d)	(a + b + c + d)

CONFIDENTIAL

TABLE 5 DEFINITION OF PERFORMANCE METRICS

a = True Positives,<br>b = False Positives<br>c = False Negatives<br>d = True Negatives	Sensitivity = a / (a + c)<br><br>Specificity = d / (b + d)

The diagnostic yield for a test is defined as the proportion of confirmed TB cases (based on the eMRS result) that also tested positive by the test. The diagnostic yield therefore can be considered a combination of the ability of the patient to provide a sample and the performance of a test on that sample. The estimate will be calculated together with 95% confidence intervals based on Wilson's score method.

<u>Primary endpoints</u>

Endpoints 1.1 and 1.2

Point estimates of sensitivity and specificity will be derived for FujiLAM using an extended microbiological reference standard (eMRS, endpoint 1.1) and the composite reference standard (CRS, endpoint 1.2) on the PP population.

Endpoint 1.3

The point estimate of the diagnostic yield will be derived for FujiLAM, AlereLAM, Ultra sputum, Ultra urine and smear on the ITT population including participants who fall under the Unclassifiable category (Table 2).

<u>Secondary endpoints</u>

Endpoint 2.1

Endpoint 2.1 will be evaluated like endpoint 1.1. Point estimates of sensitivity and specificity, with 95% confidence intervals, will be derived for FujiLAM using a microbiological reference standard (MRS) on the PP population.

Endpoint 2.2

Endpoint 2.2 will be evaluated like endpoint 1.3 for FujiLAM, AlereLAM, Ultra sputum, Ultra urine and smear on the ITT population including participants who fall under the Unclassifiable category.

Endpoint 2.3

Point estimates of sensitivity and specificity, with 95% confidence intervals will be derived for FujiLAM using a MRS, eMRS and CRS separately on the PP population for the following subgroups:

- CD4 strata defined by cytometer (CD4≤200, CD4>200)
- CD4 strata defined by VISITECT® CD4 Advanced Disease (CD4≤200, CD4>200)
- Outpatient vs. inpatients
- by ART status (on ART, not on ART)
- PTB vs EPTB vs PTB&EPTB

CONFIDENTIAL

- number of anatomical sites with confirmed TB disease
- WHO clinical stage of HIV disease
- TB history
- enrolment sites
- WHO danger signs (respiratory rate > 30/min, heart rate > 120/min, temperature > 39 °C, and being unable to walk unaided)

Details on the definition of subgroups will be described in the SAP.

Endpoint 2.4

Point estimates of sensitivity and specificity, with 95% confidence intervals will be derived for FujiLAM using a MRS, eMRS and CRS separately on the PP population, excluding patients who do not have FujiLAM results on Day 2 urine.

Endpoint 2.5

Endpoint 2.5 will be evaluated as 2.4 for the same subgroups described for endpoint 2.3.

Endpoint 2.6

Point estimates of sensitivity and specificity, with 95% confidence intervals, will be derived for AlereLAM in the same way as described in 1.1, 1.2 and 2.1, 2.3 excluding those patients for whom AlereLAM result is not available.

Endpoint 2.7

The point estimate of the diagnostic yield, with 95% confidence interval, will be derived, as for endpoint 1.3, for FujiLAM over or in combination with Ultra sputum and/or smear on the ITT population including participants who fall under the Unclassifiable category.

The difference in sensitivity and specificity between FujiLAM and AlereLAM tests will be derived and reported, separately per each endpoint and population, with 95% confidence intervals calculated using Tango's score method.

Additional exploratory endpoints, such as operational characteristics, added value of Day 2 urine and sensitivity analyses (including unclassifiable etc.) will be detailed in the SAP.

Endpoint 2.8

Kaplan–Meier survival curves will be generated to investigate the risk of mortality at 2-3 months, based on FujiLAM test results among all patients and those with Definite TB of those sites where vital status assessment can be done.

Endpoint 2.9

Point estimates of sensitivity and specificity, with 95% confidence intervals, will be derived for VISITECT® CD4 Advanced Disease test, using cytometry as the reference standard.

The sensitivity and specificity will be calculated by first preparing 2 x 2 tables and comparing the VISITECT® CD4 Advanced Disease test results against the Flow Cytometry reference method results (**Error! Reference source not found.**, **Error! Reference source not found.**). Patients who do not have results for either the Omega test or the reference standard, will be excluded from the analysis.

FIND

TABLE 6 CONFUSION MATRIX: DEFINITION OF TEST RESULTS FOR VISITECT® CD4 ADVANCED DISEASE

		FLOW cytometry	
		≤200	>200
VISITECT® CD4 Advanced Disease	≤200	a	c
	>200	b	d

Additional exploratory endpoints such as e.g. How often does VISITECT® CD4 Advanced Disease misclassify a patient who should be identified for testing with FujiLAM (i.e. CD4 <200) as CD4>200 and thus prevent appropriate use of the FujiLAM assay, will be included in the SAP.

Endpoint 2.10

Feasibility will be assessed by asking the POC heath care worker to fill a questionnaire about design, ease of use and implementation of the FujiLAM test.

8.3 Sample Size Determination

The target sample size was chosen to achieve an acceptable level of precision for the estimates of FujiLAM sensitivity and specificity for MTB-detection in PLHIV.
It has been assumed that the average expected sensitivity for FujiLAM should be 60%, based on data previously gathered on subjects with CD4 count below 200 [cell/ul] (sensitivity of 75%) and above 200 [cell/ul] (sensitivity of 45%). Based on these considerations, a sample size of 233 Confirmed TB patients to be enrolled was determined as adequate to obtain an estimate of the expected sensitivity with a precision of +/-L = 9%, at a significance level alpha = 0.05 (corresponding to 95% confidence interval), power 0.8 (probability that the width of the confidence interval is 2L or less), and power to detect the required number of cases of 0.8[5].
Considering 20% of Lost to follow up cases the total sample size to be enrolled would be dependent on the average TB prevalence (Table 7). Study enrolment will continue until the determined 233 confirmed TB cases are reached.

TB prevalence %	FujiLAM sensitivity (%)	Precision (%)	α	Power	Confirmed TB[¢]	Total sample size (N)	N with 20% LTFU
20	60	9	0.05	0.8	233	1200	1440
15	60	9	0.05	0.8	233	1650	1980

TABLE 7 SAMPLE SIZE CALCULATION DEPENDENT ON THE PREVALENCE OF TB

[¢]The number of Confirmed TB cases will be assessed during enrolment with the minimal criteria of having MRS test results available (including culture)

CONFIDENTIAL

8.4 Other analyses

Additional and exploratory analyses will be specified in the SAP.

8.5 Planned Interim Analyses

No interim analysis will be done in this study.

8.6 Statistical software

The data analysis will be performed using the R statistical software, version 3.4 or higher, and Microsoft Excel version 16.16 or higher for initial visualization of the data.

9 Regulatory and Ethical Considerations

9.1 Regulatory and Ethics Approvals

This trial will be conducted in accordance with the protocol and with the following:

- Consensus ethical principles derived from international guidelines including the Declaration of Helsinki
- Applicable Good Clinical Practice Guidelines: ICH GCP E6 (R2)
- Applicable laws and regulations

The protocol, protocol amendments, ICF and other relevant documents (e.g. advertisements) must be submitted to an IRB/IEC by the investigator and reviewed and approved by the IRB/IEC before the trial is initiated. A copy of the IRB/IEC approval letter will be filed in the investigator site file.

FIND-approved versions of an amended trial protocol must be signed by the Investigator(s). Any substantial amendments to the protocol will require IRB/IEC approval before implementation of changes made to the trial design, except for changes necessary to eliminate an immediate hazard to trial participants. Protocol amendments restricted to clerical edits only will be provided to the trial sites and submitted to the IRB/IEC for informational purposes.

The investigator will be responsible for the following:

- Providing written summaries of the status of the trial to the IRB/IEC annually or more frequently in accordance with the requirements, policies, and procedures established by the IRB/IEC
- Notifying the IRB/IEC of SAEs or other significant safety findings as required by IRB/IEC procedures
- Providing oversight of the conduct of the Trial at the site and adherence to requirements of ICH guidelines, the IRB/IEC, the WHO Good Clinical Laboratory Practice (GCLP), and with applicable national regulations.

Before the start of the trial, the investigators will provide the following documents:

- Curriculum vitae of the Principal Investigator (PI)
- Protocol Signature Page, signed and dated by the PI
- Financial Disclosure Form(s) completed by the PI and sub-investigators, if applicable (see section 9.2 Financial Disclosure)
- IRB/IEC approval letter for the trial protocol and consent form.

A protocol deviation is an unplanned excursion from, or an instance of, non-compliance with the protocol as written. All protocol deviations shall be documented and reported to FIND trial staff according to the Trial Manual and, if required, to the trial site's IRB/IEC. Each protocol deviation and its assessed impact on trial data will be described in the clinical trial report.

9.2 Financial Disclosure

Investigators and sub-investigators will provide FIND with sufficient, accurate financial information as requested to allow FIND to submit complete and accurate financial certification or disclosure statements to the appropriate regulatory authorities.

9.3 Informed Consent Process

The investigator or his/her legally authorized representative will explain the nature of the trial to the potential participant and answer all questions regarding the trial.

Potential trial participants must be informed that their participation is voluntary. Participants will be required to sign and date a statement of informed consent that meets the requirements of local regulations and/or ICH guidelines where applicable, and the IRB/IEC or trial centre. The consent forms will describe in detail the trial procedures and risks/benefits associated with participation in the trial. The rights and welfare of potential subjects will be protected by emphasizing that neither their access to medical care nor the quality of their care will be adversely affected if they decline to participate in this trial.

There must be evidence that written informed consent was obtained before the participant was enrolled in the trial and ample time was given to participant to consent. The date the written consent was obtained (as well as the time, ideally) must be recorded. The authorized person obtaining the informed consent must also sign and date the ICF. A copy of the ICF(s) must be provided to the participant.

Illiterate participants must provide a thumbprint on the ICF and the ICF signed and dated by an impartial witness.

Participants must be re-consented to the most current version of the ICF(s) during their participation in the trial, if required.

A copy of the ICF(s) will be given to the participant or the participant's legally authorized representative.

The ICF will contain a separate part that addresses the use of remaining urine samples for optional exploratory research as well as storage of chest X-rays in FIND's databank. The investigator or

CONFIDENTIAL

authorized designee will explain to each participant the objectives of the exploratory research. Participants will be told that they are free to refuse to participate and may withdraw their consent at any time and for any reason during the storage period.

9.4 Data Protection

Participants will be assigned a unique identifier generated by FIND. Any participant records or datasets that are transferred to FIND will contain the identifier only; participant names or any information which would make the participant identifiable will not be transferred.

The participant will be informed that his/her personal trial-related data will be used by FIND in accordance with local data protection law. The level of disclosure must also be explained to the participant.

The participant will be informed that his/her medical records may be examined by quality assurance auditors or other authorized personnel appointed by FIND, by appropriate IRB/IEC members, and by inspectors from regulatory authorities.

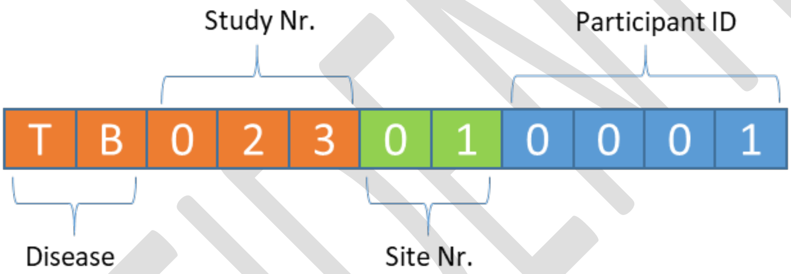

Figure 8 Example of the Unique Identifier

10 Data Handling and Record Keeping

FIND is responsible for the data management of this trial including quality control checks of the data and assessment of overall protocol compliance. All participant data relating to the trial will be recorded in source documents and transcribed on to a paper or electronic Case Report Form (CRF) by trial site staff. Data will then be entered from the paper CRF into FIND's online clinical trials platform (OpenClinica Enterprise Edition *version 4.0*), unless transmitted to FIND electronically (e.g., direct electronic data capture (EDC) or transfer of laboratory electronic datasets). The investigator is responsible for verifying that data entries are accurate and correct by electronically signing the CRF.

Records and documents, including signed ICFs, pertaining to the conduct of this trial must be retained by the investigator for 10 years after trial completion unless local regulations or institutional policies require a longer retention period. No records may be destroyed during the retention period without the written approval of FIND. No records may be transferred to another location or party without written notification to FIND.

10.1 Source Data and Source Documents

Source documents provide evidence for the existence of the participant and substantiate the integrity of the data collected. The investigator/institution should maintain adequate and accurate source documents and trial records that include all pertinent observations on each of the site's trial participants (source data). The investigator may need to request previous medical records or reports (if available), depending on the trial. Source documents are filed at the investigator's site.

Source data should be attributable, legible, contemporaneous, original, accurate and complete. Changes to source data should be traceable, should not obscure the original entry and should be explained if necessary.

The definition of what constitutes source data can be found in Table 8.

The investigator must permit trial-related monitoring, audits, IRB/IEC review, and regulatory agency inspections and provide direct access to participant medical records and source documents used for this trial.

Participant data will consist of medical history, clinical examination, laboratory testing and FU information. Observations from the clinical examination will be recorded directly in the eCRF.

**Type of source data**	
**Demographics**	**eCRF/pCRF**
**Medical history**	**eCRF/pCRF**
**Patient questionnaire**	**eCRF/pCRF**
**Specimen collecting time and date**	**eCRF/pCRF**
**Chest Xray**	**Xray report**
**Lab results**	**Lab notebook**

TABLE 8 SOURCE DATA DEFINITION AND RECORD

Other unidentified source data will be described in the Site Initiation Visit Report. Lab staff authorized by the PI will be trained and given a unique a password to enter lab data originally recorded in laboratory notebooks directly into the electronic CRF. Thereby avoiding time consuming transcription on to a paper CRF first.

10.2 Data Management

Data Management procedures at FIND, including the setup of the database, programming edit and range checks and querying, are described in the Data Management Plan.

Whenever possible clinical data and laboratory results will be captured directly onto electronic CRF designed by FIND in the OpenClinica EDC system. Site staff will be responsible for entering their

CONFIDENTIAL

data from a paper CRF into OpenClinica where direct capture into EDC is not possible. Detailed timelines for data transfer will be provided in the Trial Manual. Data will be cleaned of errors by FIND throughout the trial as it is captured electronically.

The investigator is responsible for verifying that data entries are accurate and correct. Data entered in the OpenClinica database must be consistent with the source documents or the discrepancies must be explained.

The site will be provided with individual password-protected accounts to access OpenClinica, following a training session given by FIND.

OpenClinica provides an audit trail system recording all data entries/changes and queries between FIND and the site. Data entry training will be provided by FIND, either on site or remotely sharing screen through Skype or any other similar system.

No information concerning the trial or the data generated from the trial will be released to any unauthorized third party without prior written approval of FIND.

11 Quality Management

Quality Management for this trial consists of Quality Control activities, training and capacity building provided by FIND (or designee) to the investigational sites and laboratories, as well as the use of Standard Operating Procedures, Work Instructions, Tools and Templates.

Training on the protocol, GCP and the use of the IVDs and laboratory tests will be provided by FIND. A Trial Manual, which describes all of the sample testing procedures, will be provided by FIND prior to the commencement of the Trial. Training on the EDC system will be provided by FIND Data Management prior to first participant enrolment.

11.1 Quality Control (monitoring)

Quality control should be applied to each stage of data handling to ensure that all data are reliable and have been processed correctly. The investigational site is responsible for performing regular Quality Control checks on the data they generate.

FIND will perform risk based monitoring of this trial, and associated Quality Control checks, as described in the Monitoring Plan. Trial monitors will perform source data review and source data verification to confirm that data entered into the CRF by authorized site personnel are accurate, complete, and verifiable from source documents; that the safety and rights of participants are being protected; and that the trial is being conducted in accordance with the currently approved protocol and any other trial agreements, ICH GCP, and all applicable regulatory requirements.

11.2 Quality Assurance (auditing)

As part of routine Quality Assurance, FIND or designee may conduct an audit of the investigational site.

CONFIDENTIAL

Regulatory authorities may also perform an inspection. Investigators should contact FIND staff immediately upon notification of an inspection.

11.3 Trial and Site Closure

FIND reserves the right to close the trial site or terminate the trial at any time for any reason at its sole discretion. Investigational sites will be closed upon trial completion. A trial site is considered closed when all required documents and trial supplies have been collected and participant samples shipped to the FIND biorepository (if needed).

The investigator may initiate trial-site closure at any time, provided there is reasonable cause and sufficient notice is given in advance of the intended termination.

Reasons for early closure of a trial site by FIND are described in the contractual agreement.

12 Publication Policy

Data obtained from participation in this trial are considered confidential and may be used to support market approval. The investigators must adhere to the non-disclosure requirements set forth in the FIND agreement.

The investigator is obligated to provide FIND or its designee with complete test results and all data obtained in this trial. Only FIND may make information obtained during this trial available to other investigators and third parties, including regulatory agencies.

Authorship for scientific publication of the trial results will be determined by mutual agreement and in line with International Committee of Medical Journal Editors authorship requirements, as described in the publication policy section of the contractual agreement.

13 References

---

[1] World Health Organization. Global Tuberculosis Report 2017. (2017). doi:WHO/HTM/TB/2017.23

[2] Getahun, H. & Ford, N. Tackling the persistent burden of tuberculosis among people living with HIV. J. Int. AIDS Soc. 19, 21002 (2016).

[3] Broger T. et al., Novel High Sensitivity Tuberculosis Point-of-Care Test for People Living with HIV (September 21, 2018). Available at SSRN: https://ssrn.com/abstract=3254479

[4] Gina P. et al., Early morning urine collection to improve urinary lateral flow LAM assay sensitivity in hospitalised patients with HIV-TB co-infection. BMC Infect Dis., 2017; 17: 339.

[5] Chapter 6, Zhou et al, Statistical Methods in Diagnostic Medicine, Second Edition, Wiley 2011

FIND

14 Appendices

Appendix 1: Safety Definitions and Reporting

Adverse Event (AE) Definition
• An AE is any unfavourable and unintended sign (including abnormal laboratory finding), symptom or disease temporally associated with the use of an investigational product, whether or not related to the investigational product.

Serious Adverse Event (SAE) Definition:
a. Results in death
b. Is life-threatening<br>The term 'life-threatening' in the definition of 'serious' refers to an event in which the participant was at risk of death at the time of the event. It does not refer to an event, which hypothetically might have caused death, if it were more severe.
c. Requires inpatient hospitalization or prolongation of existing hospitalization<br>In general, hospitalization signifies that the participant has been detained (usually involving at least an overnight stay) at the hospital or emergency ward for observation and/or treatment that would not have been appropriate in the physician's office or outpatient setting. Complications that occur during hospitalization are AEs. If a complication prolongs hospitalization or fulfils any other serious criteria, the event is serious. When in doubt as to whether "hospitalization" occurred or was necessary, the AE should be considered serious.<br>Hospitalization for elective treatment of a pre-existing condition that did not worsen from baseline is not considered an AE.
d. Results in persistent disability/incapacity<br>• The term disability means a substantial disruption of a person's ability to conduct normal life functions.<br>• This definition is not intended to include experiences of relatively minor medical significance such as uncomplicated headache, nausea, vomiting, diarrhea, influenza, and accidental trauma (e.g., sprained ankle) which may interfere with or prevent everyday life functions but do not constitute a substantial disruption.
e. Is a congenital anomaly/birth defect
f. Other situations:<br>• Medical or scientific judgment should be exercised in deciding whether SAE reporting is appropriate in other situations such as important medical events that may not be immediately life-threatening or result in death or hospitalization but may jeopardize the participant or may require medical or surgical intervention to prevent one of the other outcomes listed in the above definition. These events should usually be considered serious.<br>Examples of such events include intensive treatment in an emergency room or at home for allergic bronchospasm or convulsions that do not result in hospitalization, or development of drug dependency or drug abuse.

CONFIDENTIAL

*For the purpose of this study only SAEs which may be related to study procedures will be reported to FIND. All SAEs should be reported to local regulatory bodies as per local/national guidelines.

**SAE Reporting to FIND**
• The SAE Report must be sent to the FIND Head of Program and Trial Manager via e-mail, marked High Priority, with a follow up call to ensure receipt.<br>• Initial notification via telephone does not replace the need for the investigator to complete and sign the SAE Report within the designated reporting time frames.<br>• Contacts for SAE reporting can be found on the front of the protocol.

CONFIDENTIAL

Appendix 2: Incident Definition and Reporting

Incident: Any malfunction or deterioration in the characteristics and/or performance of a device, as well as any inadequacy in the labeling or the instructions for use which, directly or indirectly, might lead to or might have led to the death of a patient, or USER or of other persons or to a serious deterioration in their state of health.

The following criteria as defined in sections 1.1, 1.2 and 1.3 are to be considered to determine whether an adverse incident has occurred. Please note that **all three criteria** must be met to be deemed as reportable. Please also refer to MEDDEV 2.12-1, Guidelines on a Medical Devices Vigilance System for further guidance.

1.1 An Event has Occurred

This also includes situations where testing performed on the device, examination of the information supplied with the device or any scientific information indicates some factor that could lead or has led to an event.

Typical events include, but are not limited to:

1.1.1 A malfunction or deterioration in the characteristics or performance (should be for intended purpose and follow manufacturer's instructions).

1.1.2 For IVDs where there is a risk that an erroneous result would either (1) lead to a patient management decision resulting in an imminent life-threatening situation to the individual being tested, or to the individual's offspring, or (2) cause death or severe disability to the individual or foetus being tested, or to the individual's offspring, all false positive or false negative test results shall be considered as events.

For all other IVDs, false positive or false negative test result falling outside the declared performance of the test shall be considered as events.

1.1.3 Unanticipated adverse reaction or side effect.

1.1.4 Interactions with other substances or products.

1.1.5 Degradation / destruction of the device.

1.1.6 Inappropriate therapy.

1.1.7 Inaccurate labelling, Instructions for Use (IFU) and / or promotional materials (includes omissions and deficiencies but not those generally known by user).

1.2 The Manufacturer's Device is Suspected to be a Contributory Cause of the Incident

1.2.1 In assessing the link between the device and the Incident, the Manufacturer should take account of:

1.2.1.1 Results of the manufacturer's own preliminary assessment of the incident.

CONFIDENTIAL

1.2.1.2 Evidence of previous, similar incidents.

1.2.1.3 Any other relevant evidence held by the manufacturer.

1.2.2 Where there are a number of devices involved, the manufacturer should always assume their device contributed to the incident until proven otherwise.

1.3 The Event Led, or Might Have Led, to One of the Following:

1.3.1 The death of a patient, user or other person.
1.3.2 Serious deterioration in the health of a patient, user or other person that can include:
1.3.3 Life-threatening illness.
1.3.4 Permanent impairment of a body function or permanent damage to a body structure.
1.3.5 A condition necessitating medical or surgical intervention to prevent the two points above; for example clinically relevant increase in surgical procedure, a condition requiring hospitalisation or prolongation of existing hospitalisation.
1.3.6 Any indirect harm as a consequence of incorrect diagnostic or IVD test result when used within the manufacturer's IFU (use errors reportable under section 5.1.5.1 of MEDDEV 2.12/1 must also be considered)
1.3.7 Foetal distress, foetal death or any congenital abnormality or birth defect.

CONFIDENTIAL
