# Supplementary material for: Urine-Xpert Ultra for the diagnosis of tuberculosis in people living with HIV: a prospective, multicentre, diagnostic accuracy study
Source: Lancet Glob Health. 2024 Nov 20;12(12):e2024–2034. doi: 10.1016/S2214-109X(24)00357-7 (PMC11584317; doi:10.1016/S2214-109X(24)00357-7)
Supplement: Equitable Partnership Declaration [file mmc2.pdf]

THE LANCET
Global Health

Supplementary appendix 2

This Equitable Partnership Declaration (EPD) was submitted by the authors, and we reproduce it as supplied. It has not been peer reviewed. *The Lancet*'s editorial processes have not been applied to the EPD.

Supplement to: Sossen B, Székely R, Mukoka M, et al. Urine-Xpert Ultra for the diagnosis of tuberculosis in people living with HIV: a prospective, multicentre, diagnostic accuracy study. *Lancet Glob Health* 2024; **12:** e2024–34.

Equitable Partnership Declaration questions

This Equitable Partnership Declaration is a statement being published online alongside papers at *The Lancet Global Health*, as a separate appendix, to allow researchers to describe how their work engages with researchers, communities, and environments in the countries of study. This is part of our broader goal to decolonise global health, handing control and leadership of research to academics and clinicians who are based in the regions of study, and to affected communities.

Please answer all questions with as much detail as possible, noting that all included information will be published open-access and it will be freely available online to all who wish to read it. If a question does not apply to your study, please state "Not applicable".

The format of and questions in this statement are currently in a pilot phase. Please email Dr Kate McIntosh (Kate.McIntosh@lancet.com; deputy editor) with any feedback, particularly if you find any questions unclear.

Researcher considerations

1. Please detail the involvement that researchers who are based in the region(s) of study had during a) study design; b) clinical study processes, such as processing blood samples, prescribing medication, or patient recruitment; c) data interpretation; and d) manuscript preparation, commenting on all aspects. If they were not involved in any of these aspects, please explain why.

   This question is intended for international partnerships; if all your authors are based in the area of study, this question is not applicable.

   This should include a thorough description of their leadership role(s) in the study. Are local researchers named in the author list or the acknowledgements, or are they not mentioned at all (and, if not, why)? Please also describe the involvement of early career researchers based in the location of the study. Some of this information might be repeated from the Contributors section in the manuscript. Note: we adhere to ICMJE authorship criteria when deciding who should be named on a paper.

**a) Study design:**<br>N/A - *authors are based in the area of study*
**b) Clinical study processes:**<br>N/A - *authors are based in the area of study*
**c) Data interpretation:**<br>N/A - *authors are based in the area of study*
**d) Manuscript preparation:**<br>N/A - *authors are based in the area of study*

2. Were the data used in your study collected by authors named on the paper, or have they been extracted from a source such as a national survey? ie, is this a secondary analysis of data that were

not collected by the authors of this paper. If the authors of this paper were not involved in data collection, how were data interpreted with sufficient contextual knowledge?

The Lancet Global Health *believe contextual understanding is crucial for informed data analysis and interpretation.*

Data was collected prospectively by named authors

3. How was funding used to remunerate and enhance the skills of researchers and institutions based in the area(s) of study? And how was funding used to improve research infrastructure in the area of study?

   Potentially effective investments into long-term skills and opportunities within institutions could include training or mentorship in analytical techniques and manuscript writing, opportunities to lead all or specific aspects of the study, financial remuneration rather than requiring volunteers, and other professional development and educational opportunities.

   Improvements to research infrastructure could be funding of extended trial designs (such as platform trials) and use of master protocols to enable these designs, establishment of long-term contracts for research staff, building research facilities, and local control of funding allocation.

**Skills:**<br><br>Local students are gaining skills and experience, such as towards PhD projects.
**Research infrastructure:**<br><br>Local sites were each allocated funding independently by the sponsor.

4. How did you safeguard the researchers who implemented the study?

   Please describe how you guaranteed safe working conditions for study staff, including provision of appropriate personal protective equipment, protection from violence, and prevention of overworking.

Authors are from the area of study and this work was conducted in their usual/routine work environments

Benefits to the communities and regions of study

5. How does the study address the research and policy priorities of its location?

   How were the local priorities determined and then used to inform the research question? Who decided which priorities to take forward? Which elements of the study address those priorities?

   Based on local and international priorities for rapid diagnostic tools for tuberculosis.

6. How will research products be shared in the community of study?

   For instance, will you be providing written or oral layperson summaries for non-academic information sharing? Will study data be made available to institutions in the region(s) of study? The Lancet Global Health *encourages authors to translate the summary (abstract) into relevant languages after paper editing; do you intend to translate your summary?*

   Our work includes teams from 7 different countries, which all speak multiple languages therein: for example there are 11 official languages in South Africa, 3 of which are commonly spoken in the province where this study occurred. It will not be feasible, and it will be very expensive, to translate our summary into all of these relevant languages.

   In Malawi:
   Study results are presented to the community advisory board at the team's institution as well as to the National TB and Leprosy programmes.

   In South Africa:
   Study results will be disseminated to local communities and stakeholders via social media, at research forums, and at the National TB Think Tank.

   In Tanzania:
   The team plan on disseminating study findings via technical working groups and the research community, within the planned meetings that are organised by the TB and HIV control programmes. The communication team will also share the study findings locally, in lay language, through official institutional social media platforms and their monthly online magazine.

   In Thailand:
   The team plan on disseminating study findings in technical working groups and planned meetings of the TB and HIV control programmes as well as the advanced HIV disease programme. In the lectures given to local healthcare workers and communities on HIV and TB, the relevant areas will now be updated. The findings will also be shared locally, in lay language, via the institute's website.

<u>In Uganda:</u><br>The team disseminates results via the institutional research forums and conferences.<br><br><u>In Vietnam:</u><br>The team plan on disseminating results via local conferences and social media, including via translations of relevant areas of lectures.<br><br><u>In Zambia:</u><br>The team plan on disseminating locally via the TB technical working group and the research community. PowerPoint presentations will be formatted and articulated with lay language, appropriate for community level.

7. How were individuals, communities, and environments protected from harm?

a) *How did you ensure that sensitive patient data was handled safely and respectfully? Was there any potential for stigma or discrimination against participants arising from any of the procedures or outcomes of the study?*

Informed consent and review process happened in a private area and all captured data was de-identified. Work was conducted and co-authored by local team members, who were sensitive to avoiding stigma and discrimination based on locally-appropriate measures. Research was conducted in line with Good Clinical Practice guidelines.

b) *Might any of the tests be experienced as invasive or culturally insensitive?*

No

c) *How did you determine that work was sensitive to traditions, restrictions, and considerations of all cultural and religious groups in the study population?*

Work was conducted and co-authored by local team members, who were sensitive to these.

d) *Were biowaste and radioactive waste disposed of in accordance with local laws?*

Yes

e) *Were any structures built that would have impacted members of the community or the environment (such as handwashing facilities in a public space)? If so, how did you ensure that you had appropriate community buy-in?*

No, not applicable

f) *How might the study have impacted existing health-care resources (such as staff workloads, use of equipment that is typically employed elsewhere, or reallocation of public funds)?*

N/A

8. Finally, please provide the title (eg, Dr/Prof, Mr/Mrs/Ms/Mx), name, and email address of an author who can be contacted about this statement. This can be the corresponding author.

**Name:** Dr Bianca Sossen<br>**Email:** bianca.sossen@uct.ac.za
